# Correlation between gut microbiome and cognitive impairment in patients undergoing peritoneal dialysis

**DOI:** 10.1186/s12882-023-03410-z

**Published:** 2023-12-05

**Authors:** Jingjing Wang, Siyang Wu, Jin Zhang, Yuanyuan Li, Yonggui Wu, Xiangming Qi

**Affiliations:** 1 Department of Nephropathy, The First Affiliated Hospital of Anhui Medical University, Hefei, 230032 Anhui PR China; 2 Center for Scientific Research of Anhui Medical University, Hefei, 230022 Anhui PR China

**Keywords:** Peritoneal dialysis, Cognitive impairment, Gut microbiome, 16S rRNA

## Abstract

**Background:**

Growing evidence has demonstrated that patients undergoing peritoneal dialysis (PD) are more likely to experience cognitive impairment than patients with non-dialysis end-stage renal disease (ESRD); however, the underlying mechanisms remain unclear. This study aimed to identify the role and predictive significance of gut microbiome alterations in PD-associated cognitive impairment.

**Methods:**

A total of 29 non-dialysis ESRD patients and 28 PD patients were enrolled in this study and divided into subgroups according to the Montreal Cognitive Assessment (MoCA). Faecal samples were analyzed using 16 S rRNA. Mini-Mental State Examination (MMSE) and MoCA scores were used to assess the degree of cognitive impairment in patients.

**Results:**

The 16 S rRNA analysis demonstrated differences in gut microbiome abundance and structure between PD and non-dialysis ESRD patients and between PD patients with cognitive impairment (PCI) and PD patients with normal cognition (PNCI). At family and genus levels, *Prevotellaceae* exhibited the greatest structure difference, while *Lactobacillus* exhibited the greatest abundance difference between PCI and PNCI. Altered microbiota abundance significantly correlated with cognitive function and serum indicators in PD. In addition, different modules related to fatty acid, lipid, pantothenate, and coenzyme A biosynthesis, and tyrosine and tryptophan metabolism were inferred from 16 S rRNA data between PCI and PNCI. Both groups could be distinguished using models based on the abundance of *Lactobacillaceae* (Area under curve [AUC] = 0.83), *Actinomycetaceae* (AUC = 0.798), and *Prevotellaceae* (AUC = 0.778) families and *Lactobacillus* (AUC = 0.848) and *Actinomyces* (AUC = 0.798) genera.

**Conclusion:**

Gut microbiome evaluation could aid early cognitive impairment diagnosis in patients undergoing PD.

**Supplementary Information:**

The online version contains supplementary material available at 10.1186/s12882-023-03410-z.

## Background

 Peritoneal dialysis (PD) is a common therapy for end-stage renal disease (ESRD). Compared to haemodialysis, PD is characterized by simple management, high dialysis efficiency, and fewer adverse reactions, which can effectively protect the kidney function of patients and improve their quality of life [1]. PD involves home-based treatment that requires self-operation and management by patients. Therefore, cognitive function is particularly important in patients undergoing PD. In older patients with cognitive impairment, loss of executive function or memory may lead to errors in PD management, increasing the risk of PD-associated peritonitis [2]. Additionally, cognitive impairment is an independent indicator of mortality and survival in patients undergoing PD [3]. Therefore, risk factors of PD-associated cognitive impairment should be explored.

The gut microbiome is a complex ecosystem. In a healthy physiological state, a stable gut microbiome can protect peritoneal function by preventing colonization by various pathogens [4]. However, long-term adverse living habits or pathological states can impair the gut microbiome structure, which affects the internal environment and metabolism of the human body, eventually causing the occurrence and progression of various diseases [5]. Recently, increasing evidence has shown that gut microbiome disorders are strongly linked to the onset and progression of nervous system diseases, the underlying mechanism of which may be related to the ‘microbe-gut-brain’ axis [6]. This association has been reported in various conditions, including Alzheimer’s disease [7], hypertension [8], ESRD [9], and in those undergoing haemodialysis [10]. However, the relationship between cognitive impairment and the faecal microbiome in patients undergoing PD remains unclear. This study aimed to delineate novel information regarding the pathogenesis, prevention, and treatment of cognitive disorders in patients undergoing PD.

## Methods

### Study cohort and clinical data collection

This study was conducted at The First Affiliated Hospital of Anhui Medical University from November 2019 to October 2021 and was approved by the hospital Ethics Committee (approval number: PJ2022-02-54). The inclusion criteria were as follows: (1) age 18–65 years; (2) the PD group met both conditions: (a) the duration of dialysis was longer than 6 months, and no PD-related peritonitis had occurred in the last 3 months, (b) regular follow-up in the PD clinic of our hospital, receiving simultaneous nursing, exercise [11], and dietary education [12]; (3) the non-dialysis ESRD group was diagnosed according to the National Kidney Foundation guidelines [13] and had not previously received any form of dialysis; and (4) no history of kidney transplantation. Exclusion criteria included: (1) prescribed any medication known to affect the gut microbiome, including antibiotics, glucocorticoids, statins, immunosuppressive agents, prebiotic supplements, phosphorus binders, and gastrointestinal drugs, within the past 1 month; (2) history of digestive diseases; (3) acute infections or other acute illnesses; (4) history of mental illness; and (5) the presence of a severe visual or hearing impairment that would preclude assessment completion by participants; (6) other factors that may influence the gut microbiome, such as a bedridden state for more than a month.

Fasting blood samples from the patients were collected and sent to our hospital laboratory for routine blood tests, liver and kidney functions, lipid profile, complement, tumour necrosis factor-alpha and interleukin-1 beta (IL-1β) levels, and c-reactive protein (CRP) levels.

### Faecal sample collection and 16 S rRNA sequencing

According to the Kidney Disease Outcomes Quality Initiative (KDOQI)’s recommendations [14], patients were given a low-salt and low-fat diet. The faecal samples were collected the day after admission, which were stored in a refrigerator at −80 °C within 24 h of collection. After all samples were collected, they were sent to BGI (Shenzhen, China) for 16 S rRNA sequencing. Microbial community deoxyribonucleic acid (DNA) was extracted using the MagPure Stool DNA KF Kit B (Magen, Guangzhou, China) following the manufacturer’s instructions. Variable regions V3–V4 of the bacterial 16 S rRNA gene were amplified with degenerate polymerase chain reaction (PCR) primers, including 341 F (5′-ACTCCTACGGGAGGCAGCAG-3′) and 806 R (5′-GGACTACHVGGGTWTCTAAT-3′). Both forward and reverse primers were tagged with the Illumina adapter, pad, and linker sequences (Illumina Inc., San Diego, CA, USA). PCR cycling conditions were as follows: 94 °C for 3 min, 30 cycles of 94 °C for 30 s, 56 °C for 45 s, 72 °C for 45 s, and a final extension for 10 min at 72 °C. The PCR products were purified using AmpureXP beads and eluted with an elution buffer. The 16 S rRNA amplicon strategy was used to construct the library. Libraries were qualified using an Agilent 2100 Bioanalyzer (Agilent, Palo Alto, CA, USA). The validated libraries were used for sequencing on the Illumina MiSeq platform (BGI, Shenzhen, China) following the standard Illumina pipeline to generate 2 × 300 bp paired-end reads.

### Cognitive, emotional, and gastrointestinal functional assessments

Questionnaire completion was guided by two trained physicians, and the scores were determined after discussion. Global cognitive function was assessed using the mini-mental state examination (MMSE) [15] and Montreal cognitive assessment scale (MoCA) [16]. Depression status was assessed using the self-rating depression scale (SDS) [17] and Hamilton depression scale (HAMD) [18]. Anxiety status was assessed using the self-rating anxiety scale (SAS) [19] and Hamilton anxiety scale (HAMA) [20]. Gastrointestinal symptoms were assessed using the gastrointestinal symptom rating scale (GSRS) [21]. Considering that the MoCA is more sensitive in identifying mild cognitive disorder than the MMSE [22], cognitive impairment was grouped according to the MoCA score, using the method described by Yu et al. [23]. MoCA scores ≥ 26 and < 26 indicated ‘normal’ and impaired cognition, respectively. In the pre-experiments, we enrolled six patients in the non-dialysis ESRD and PD groups respectively and evaluated the average MoCA score of each patient as a calculation standard for sample size (non-dialysis ESRD group, 25.50 ± 2.43; PD group, 21.33 ± 3.78). PASS software (version 15.0.5) was used for the sample size calculation as previously described [24]. The group allocation ratio was set as non-dialysis ESRD group: PD group = 1:1. After calculation, the total number of patients should not be less than 48, and the minimum sample size for each group should not be less than 24. From November 2019 to October 2021, we enrolled 29 non-dialysis ESRD patients and 28 PD patients. After scoring, the non-dialysis ESRD group was subdivided into 17 patients with normal cognition (NCI) and 12 with cognitive impairment (CI), while the PD group was subdivided into 9 patients with normal cognition (PNCI) and 19 with cognitive impairment (PCI).

### Bioinformatics and statistical analysis

#### Bacterial microbiota composition and diversity analysis

The MicrobiomeAnalyst website (https://www.microbiomeanalyst.ca/) was used to perform microbiome composition and diversity analyses [25]. We used the data filter option on the website to exclude low-count (minimum count was specified as 4; prevalence in sample was set at 20%) and low-variance data (prevalence to remove was set at 20%, based on interquartile range). Shannon and Simpson indices were calculated to analyse alpha diversity, which indicated species richness within a sample. Principal coordinate analysis (PCoA) was performed to evaluate beta diversity, which helped assess differences in community composition between the groups. In the PCoA analysis, we used the Bray–Curtis dissimilarity method to calculate the distance matrix, and the analysis of similarities (ANOSIM) statistical method to evaluate whether there were any differences between the groups.

#### Difference analysis of gut microbiome

In order to find significant differences in the bacterial species between the groups, the Wilcoxon or Kruskal–Wallis test was performed using the R software (version 3.4.1) to compare the relative abundance of the gut microbiome. LEfSe clustering and linear discriminant analysis (LDA) were performed using the LEfSe software (https://huttenhower.sph.harvard.edu/galaxy/) to compare differences in the gut microbiome composition. An LDA score > 4 was considered a biomarker of statistical difference between the groups.

#### Correlation and functional difference analysis

The SparCC algorithm was employed to calculate correlation networks using the MicrobiomeAnalyst website [25]. The Kyoto Encyclopedia of Genes and Genomes (KEGG) and MetaCyc pathways were predicted using the PICRUSt software (http://picrust.github.io/picrust/) [26]. Significant function was determined using the R software based on the Wilcoxon or Kruskal–Wallis test.

#### Other statistical analysis

All other statistical analyses were performed using SPSS software (version 26.0). Normally distributed quantitative variables are presented as means ± standard deviations (SD) and were compared using the t-test or analysis of variance. Non-normally distributed variables are presented as medians and interquartile ranges, compared using the Mann–Whitney U or Kruskal–Wallis test. Pearson’s and Spearman’s correlation coefficients were used to determine the normally and non-normally distributed variables, respectively. The *P* value for correlation between clinical markers and gut microbiome were corrected using false discovery rate (FDR) [27]. ROC curves were constructed, and the area under the curve (AUC) values were used to evaluate performance. A hypothesis diagram was constructed using the BioRender website (https://app.biorender.com/). Graphing was performed using GraphPad Prism (version 8.0.0) or the R software. Statistical significance was set at *P* < 0.05.

## Results

### Demographic patient characteristics

Of the 57 patients included in this study, 28 were undergoing PD and 29 had non-dialysis ESRD. Baseline patient characteristics are presented in Table 1 and Table S10. We observed no differences in age, sex, body mass index, years of education, or the SAS, SDS, HAMA, and HAMD scores. However, the MoCA, MMSE, and GSRS scores significantly differed in the PD group (*P* < 0.05), indicating more severe cognitive impairment and gastrointestinal symptoms.

**Table 1:** The baseline characteristics of the patients

Variables	ESRD (*n* = 29)	PD (*n* = 28)	t (Z) value	*P* value
**Sex (M / F)**	16 / 13	13 / 15	-0.654	0.513
**Age (Years)**	45.24 ± 8.02	45.75 ± 11.11	-0.199	0.843
**Education (Years)**	8.00 (6.50, 11.50)	8.00 (6.00, 8.00)	-1.470	0.141
**BMI (kg/m** ^**2**^ **)**	23.45 ± 3.47	21.82 ± 2.95	1.905	0.062
**eGFR [ml/min·1.73 m** ^**2**^ **]**	7.00 (9.00, 6.00)	4.00 (4.75, 3.00)	-5.143	0.000
**MoCA**	25.41 ± 2.95	22.43 ± 4.78	2.827	0.007
**MMSE**	29.00 (27.00, 29.00)	27.00 (24.25, 28.75)	-2.223	0.026
**SAS**	34.00 (30.50, 34.50)	34.50 (30.25, 39.00)	-1.935	0.053
**SDS**	38.93 ± 8.20	36.96 ± 8.57	0.886	0.380
**HAMA**	10.45 ± 5.08	8.54 ± 5.00	1.432	0.158
**HAMD**	6.93 ± 4.30	5.86 ± 3.59	1.022	0.311
**GSRS**	21.00 (19.00, 24.50)	23.00 (20.25, 26.00)	-2.095	0.036

Notes: The details of the patients are provided in Table S10

*Abbreviations*: *ESRD *End stage renal disease, *PD *Peritoneal dialysis, *BMI *Body mass index, *eGFR *Estimated glomerular filtration rate, *MoCA *Montreal cognitive assessment scale, *MMSE *Mini-mental state examination, *SAS *Self-rating anxiety scale, *SDS *Self-rating depression scale, *HAMA *Hamilton anxiety scale, *HAMD *Hamilton depression scale, *GSRS *Gastrointestinal symptom rating scale

### Composition and diversity analysis of gut microbiome

**Fig. 1:**
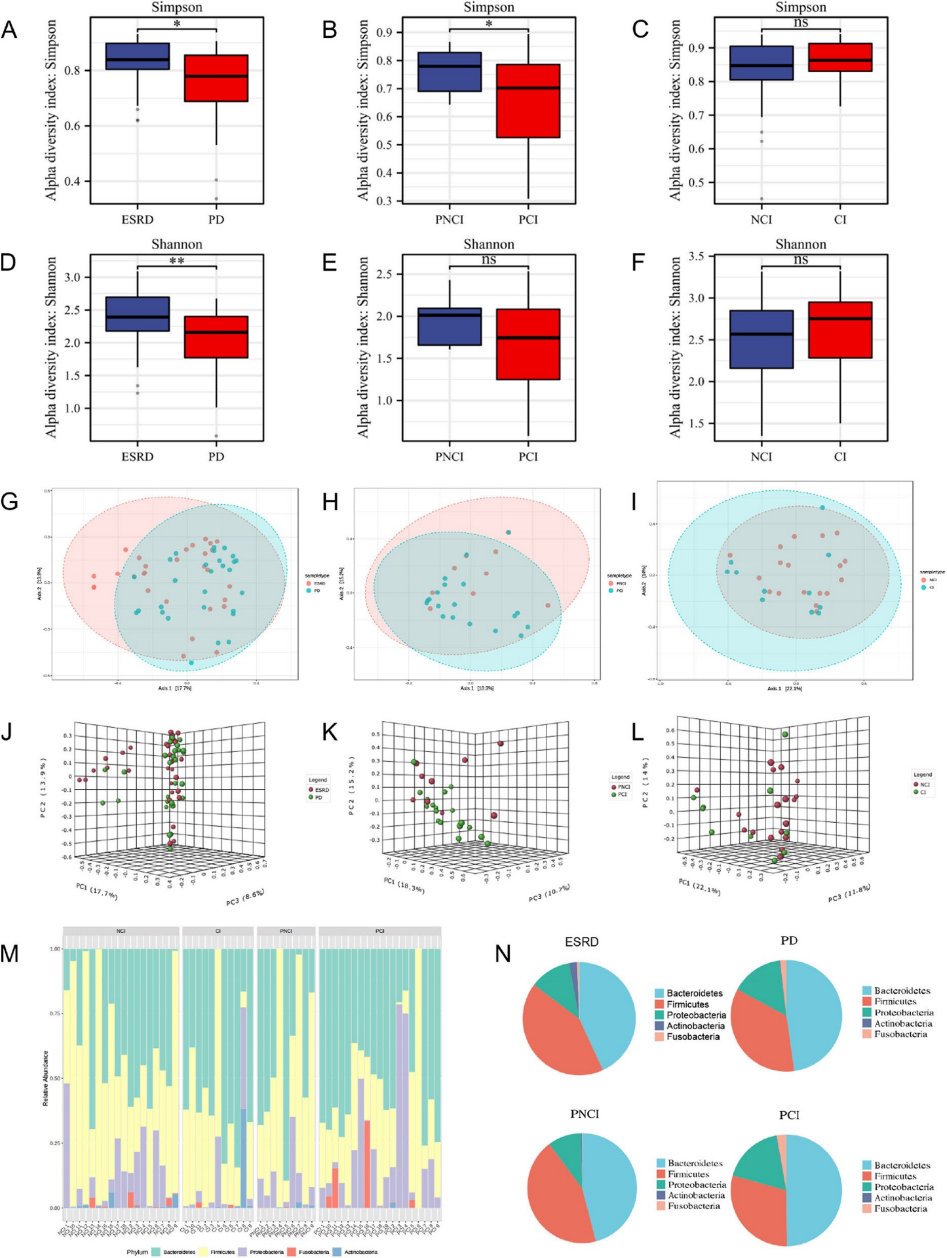
Comparison of the gut microbiome structures.Simpson indices between (**A**) non-dialysis ESRD and PD, (**B**) PNCI and PCI, (**C**) NCI and CI. Shannon indices between (**D**) non-dialysis ESRD and PD, (**E**) PNCI and PCI, (**F**) NCI and CI. Beta diversity (2D plot) between (**G**) non-dialysis ESRD and PD, (**H**) PNCI and PCI, (**I**) NCI and CI. Beta diversity (3D plot) between (**J**) non-dialysis ESRD and PD, (**K**) PNCI and PCI, (**L**) NCI and CI.** M** Stacked bar chart for relative abundance in the phylum classification. **N** Pie chart for the proportion of gut microbiome in the phylum classification. *P < 0.05, **P < 0.01. ESRD, end-stage renal disease; PD, peritoneal dialysis; CI, cognitive impairment; NCI, normal cognition; PCI, peritoneal dialysis patients with cognitive impairment; PNCI, peritoneal dialysis patients with normal cognition

 Cumulative curve is shown in Figure S1. Simpson and Shannon indices were significantly lower in the PD group than in the non-dialysis ESRD group (Fig. 1A and D). Additionally, the richness of the gut microbiome community in PCI was significantly lower than that in PNCI (Simpson indices *P* < 0.05 (Fig. 1B and E), but not between the CI and NCI groups (both Simpson and Shannon indices *P* > 0.05 (Fig. 1C and F). Based on beta diversity, the gut microbiome community composition significantly differed between the PD and non-dialysis ESRD groups (ANOSIM: R = 0.046; *P* = 0.031) and between PCI and PNCI (ANOSIM: R = 0.202; *P* = 0.029), but not between the CI and NCI groups (ANOSIM: R = 0.020; *P* = 0.358) (Fig. 1G–L, Table S1). At the phylum level (Fig. 1M–N, Table S2), the gut microbiome of PNCI group were enriched with *Bacteroidetes* (43.84%), *Firmicutes* (45.93%), *Proteobacteria* (9.81%), *Fusobacteria* (0.07%), and *Actinobacteria* (0.35%). In contrast, the PCI group, a subsequent reduction in the abundance of *Firmicutes* (29.48%) and *Actinobacteria* (0.17%) was observed, while there was increase in the abundance of *Bacteroidetes* (49.82%), *Proteobacteria* (17.72%), and *Fusobacteria* (2.8%).

### Difference analysis of gut microbiome

**Fig. 2:**
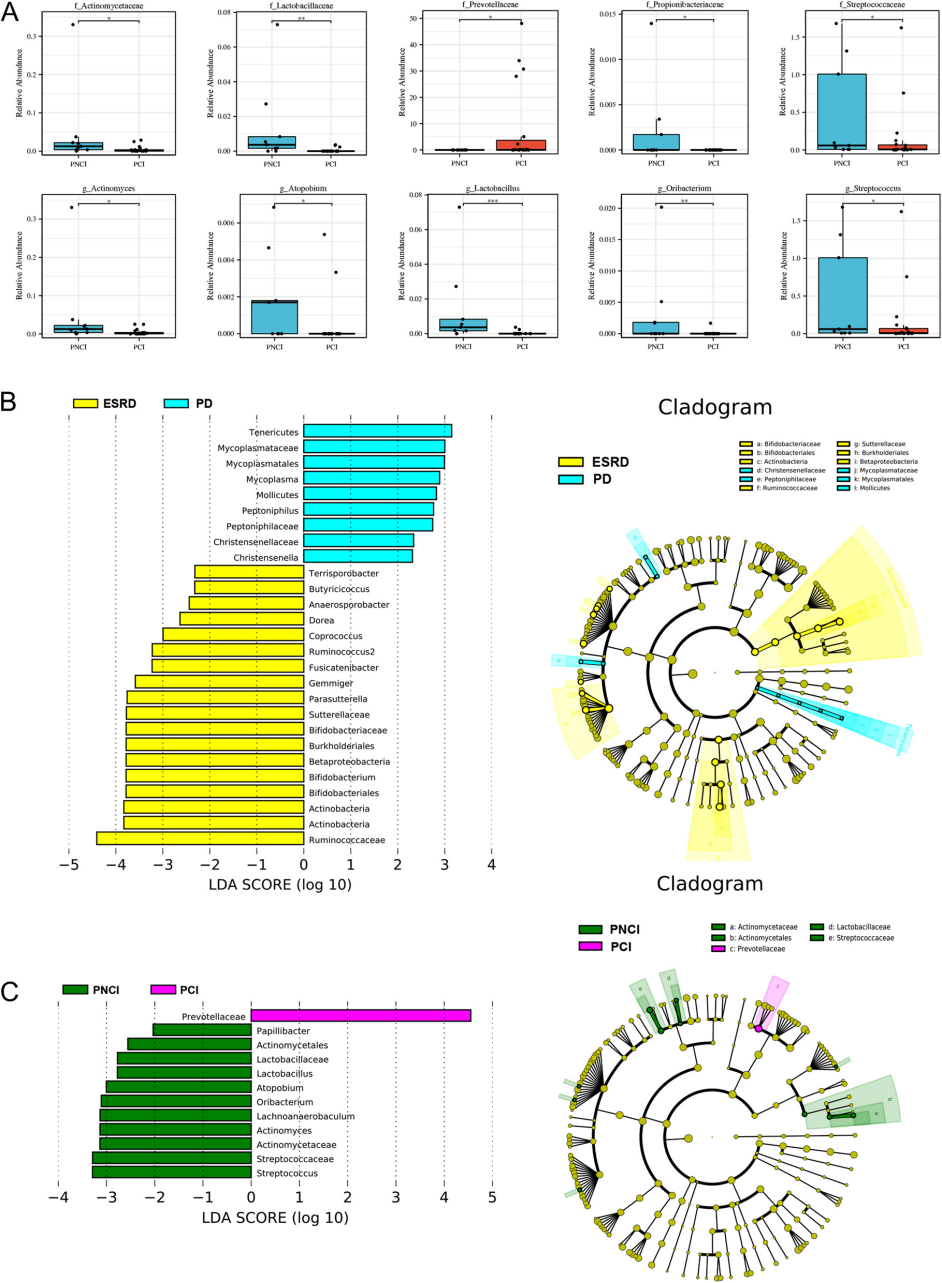
Differential analysis of gut microbiome. **A** Differences in the abundance of gut microbiome in the family and genus classifications (*P < 0.05, **P < 0.01, ***P < 0.001 vs. PNCI). Differences in the composition of gut microbiome (using Lefse analysis): (**B**) between non-dialysis ESRD and PD, (**C**) between PNCI and PCI. ESRD, end-stage renal disease; PD, peritoneal dialysis; PCI, peritoneal dialysis patients with cognitive impairment; PNCI, peritoneal dialysis patients with normal cognition

 Table 2 lists the altered microbiomes in different classifications. Compared to the ESRD group, lactic acid-producing bacteria, such as *Bifidobacterium*, short-chain fatty acid-producing bacteria, such as *Butyricicoccus*, and digestion-resistant starch bacteria, such as *Ruminococcus2*, were significantly decreased in the PD group. Compared to PNCI, lactic acid-producing bacteria, such as *Lactobacillus*, and short-chain fatty acid-producing bacteria, such as *Propionibacteriaceae* and *Clostridium butyricum*, were significantly decreased in PCI, whereas *Prevotellaceae* was significantly increased. At the family and genus levels (Fig. 2A), *Lactobacillus* showed the most obvious difference in abundance between PNCI and PCI (Wilcoxon test, *P* < 0.001). LEfSe analysis can determine the ‘value’ of each species as a discriminant group by calculating its LDA value (Fig. 2B-C). The comparison showed that *Ruminococcaceae* had a good group identification value between the non-dialysis ESRD and PD groups, whereas *Prevotellaceae* had the best identification value between PNCI and PCI (LDA > 4). More details are provided in Table S3 and S4.

**Table 2:** Gut microbiomes with differential relative abundance

Level	Change	ESRD vs. PD	PNCI vs. PCI
**Phylum**	Up	Tenericutes	/
	Down	Actinobacteria, Firmicutes	/
**Class**	Up	Mollicutes	/
	Down	Actinobacteria, Clostridia	/
**Order**	Up	Mycoplasmatales	/
	Down	Bifidobacteriales, Clostridiales	Actinomycetales
**Family**	Up	Christensenellaceae, Mycoplasmataceae, Peptoniphilaceae	Prevotellaceae
	Down	Bifidobacteriaceae, Ruminococcaceae	Actinomycetaceae, Lactobacillaceae, Propionibacteriaceae, Streptococcaceae
**Genus**	Up	Christensenella, Dialister, Mycoplasma, Olsenella, Peptoniphilus	/
	Down	Anaerosporobacter, Bifidobacterium, Butyricicoccus, Coprococcus, Dorea, Fusicatenibacter, Gemmiger, Parasutterella, Ruminococcus2, Terrisporobacter	Actinomyces, Atopobium, Lactobacillus, Oribacterium, Streptococcus
**Species**	Up	Anaerostipes_caccae, Christensenella_minuta, Clostridium_aldenense, Clostridium_ramosum, Clostridium_scindens, Eubacterium_eligens, Faecalicoccus_pleomorphus, Mycoplasma_hominis, Prevotella_timonensis	Alistipes_indistinctus, Butyricimonas_virosa
	Down	Anaerosporobacter_mobilis, Anaerostipes_hadrus, Bacteroides_massiliensis, Blautia_luti, Blautia_obeum, Blautia_schinkii, Clostridium_fimetarium, Clostridium_tarantellae, Collinsella_aerofaciens, Coprococcus_catus, Dialister_invisus, Dorea_longicatena, Fusicatenibacter_saccharivorans, Gemmiger_formicilis, Parasutterella_excrementihominis, Roseburia_inulinivorans, Ruminococcus_champanellensis, Ruminococcus_faecis, Ruminococcus_lactaris, Terrisporobacter_glycolicus	Actinomyces_dentalis, Actinomyces_odontolyticus, Atopobium_rimae, Clostridium_butyricum, Clostridium_colinum, Lachnoanaerobaculum_umeaense, Lactobacillus_fermentum, Lactobacillus_iners, Clostridium_colinum, Oribacterium_sinus, Ruminococcus_champanellensis

Notes: “Up” represents a significant increase in abundance in the PD (or PCI) group (*P* < 0.05); “Down” represents a significant decrease in abundance in the PD (or PCI) group (*P* < 0.05). The details of the results are shown in Table S3 and S4

Abbreviations: *ESRD *End stage renal disease, *PD *Peritoneal dialysis, *PNCI *Peritoneal dialysis patient with normal cognition, *PCI *Peritoneal dialysis patient with cognitive impairment

### Correlation analysis of gut microbiome at Family and Genus levels

Subsequently, we focused on correlation analysis of the altered microbiome at the family and genus levels. Microbiomes with *P* < 0.05 in the Wilcoxon test (or Kruskal–Wallis test) or LDA score > 4 were subjected to a clinical correlation analysis.

#### Gut microbiome correlation network

**Fig. 3:**
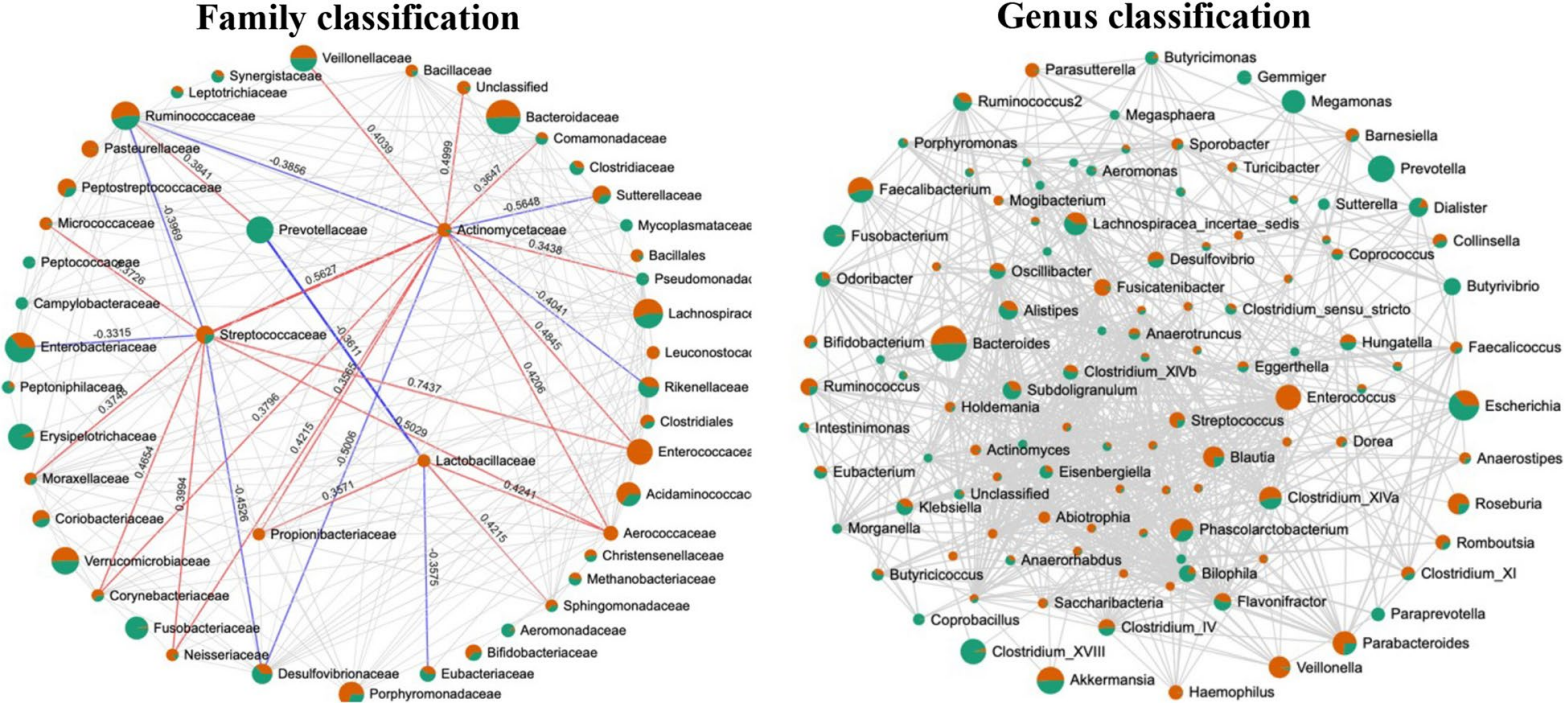
Gut microbiome correlation network for family and genus classification. The nodes that represented taxa at the family or genus level are colored according to the relative content between different groups of the classification. The green nodes represent the PCI group, whereas the red nodes represent the PNCI group. The edges represent correlations between the taxon pairs, and the number above the edge represents the correlation coefficient

 Figure 3 shows the gut microbiome correlation network between PNCI and PCI at the family and genus levels. At the family level, the altered microbiota between PNCI and PCI had potential interactions and competitive relationships with a variety of other gut bacteria. At the genus level, there existed a complex crosstalk among the species composing the gut microbiome.

#### Correlation between clinical markers and gut microbiome

**Fig. 4:**
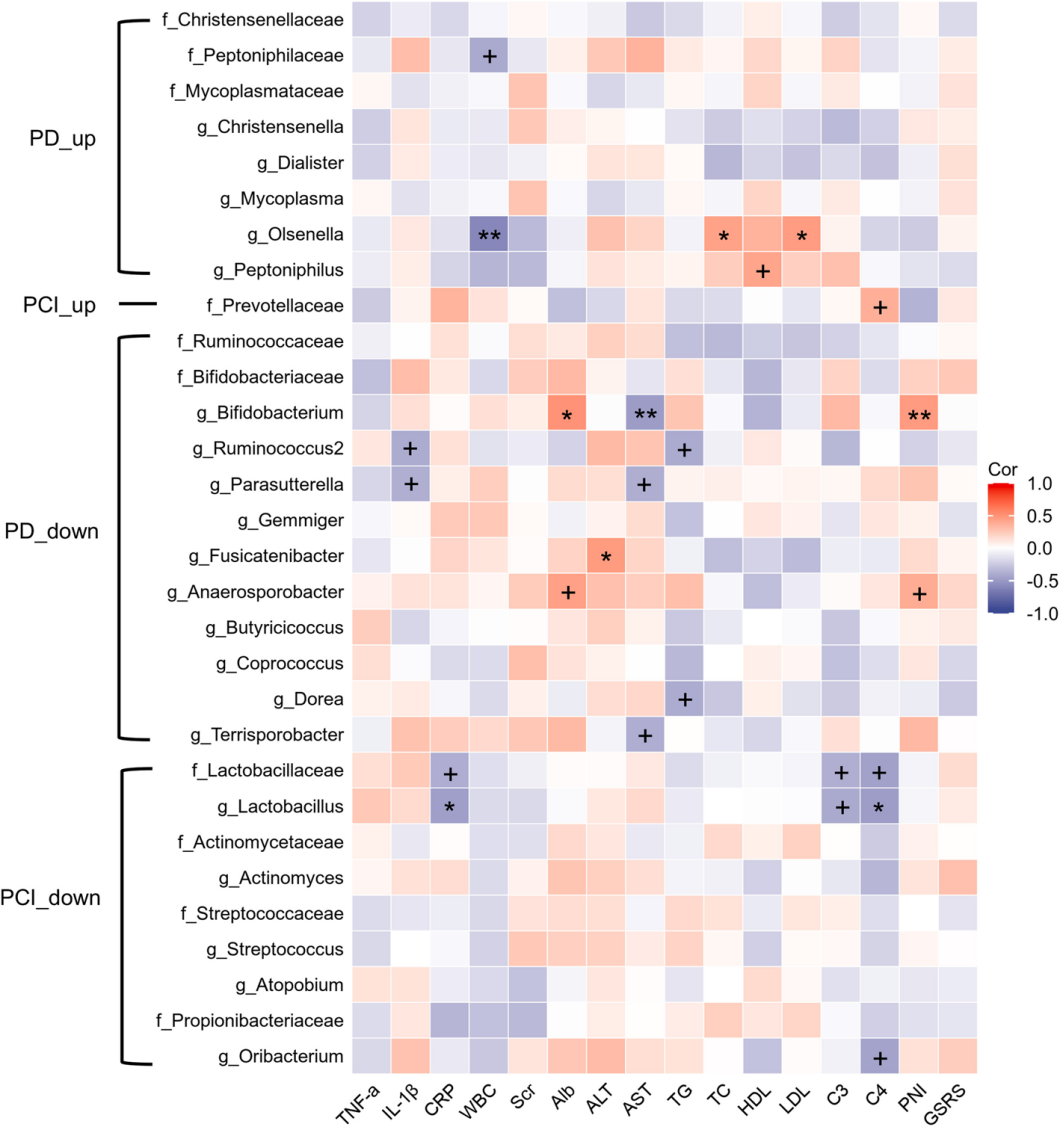
Correlation between gut microbiome and clinical markers. *FDR < 0.1, **FDR < 0.05, ^+^*FDR* > 0.1 but *P* < 0.05. PD, peritoneal dialysis; PCI, peritoneal dialysis patients with cognitive impairment; FDR, false discovery rate; WBC, white blood cell; CRP, C-reactive protein; interleukin-1beta, IL-1β; SCr, serum creatinine; Alb, albumin; ALT, alanine aminotransferase; AST, aspartate aminotransferase; TC, total cholesterol; TG, triglyceride; HDL, high-density lipoprotein; LDL, low-density lipoprotein; C3, complement-3; C4, complement-4; PNI, prognostic nutritional index; GSRS, Gastrointestinal symptom rating scale; f, family; g, genus

 In order to exclude false-positive results, we provided both *P* value and FDR in Fig. 4 and Table S5. Several significant correlations were noted between the relative abundance of the gut microbiota and clinical markers. Serum IL-1β levels were negatively correlated with *Ruminococcus2* (*P* < 0.05) and *Parasutterella* (*P* < 0.05). CRP levels were negatively correlated with *Lactobacillaceae* (*P* < 0.05) and *Lactobacillus* (FDR < 0.1). White blood cell (WBC) counts had a negative relationship with *Peptoniphilaceae* (*P* < 0.05) and *Olsenella* (FDR < 0.05). Patients with a higher serum albumin (Alb) concentration demonstrated enrichment of *Bifidobacterium* (FDR < 0.1) and *Anaerosporterbacter* (*P* < 0.05). Lipid indicators were correlated with the richness of *Ruminococcus2*, *Dorea*, and *Olsenella*. Liver functions were correlated with four microbiota (*Fusicatenibacter*, *Bifidobacterium*, *Parasutterella*, and *Terrisporobacter*). The richness of *Lactobacillaceae*, *Lactobacillus*, *Lachnoanaerobaculum*, *Oribacterium*, and *Prevotellaceae* were correlated with serum complement level. The prognostic nutritional index (PNI), which is calculated using the Alb level and peripheral lymphocyte count, is a common indicator of the nutritional status and prognosis of various diseases [28]. Our results demonstrated a positive relationship between the PNI and two microbiota (*Bifidobacterium* and *Anaerosporobacter*).

#### Correlation between cognitive or emotional scores and gut microbiome

**Fig. 5:**
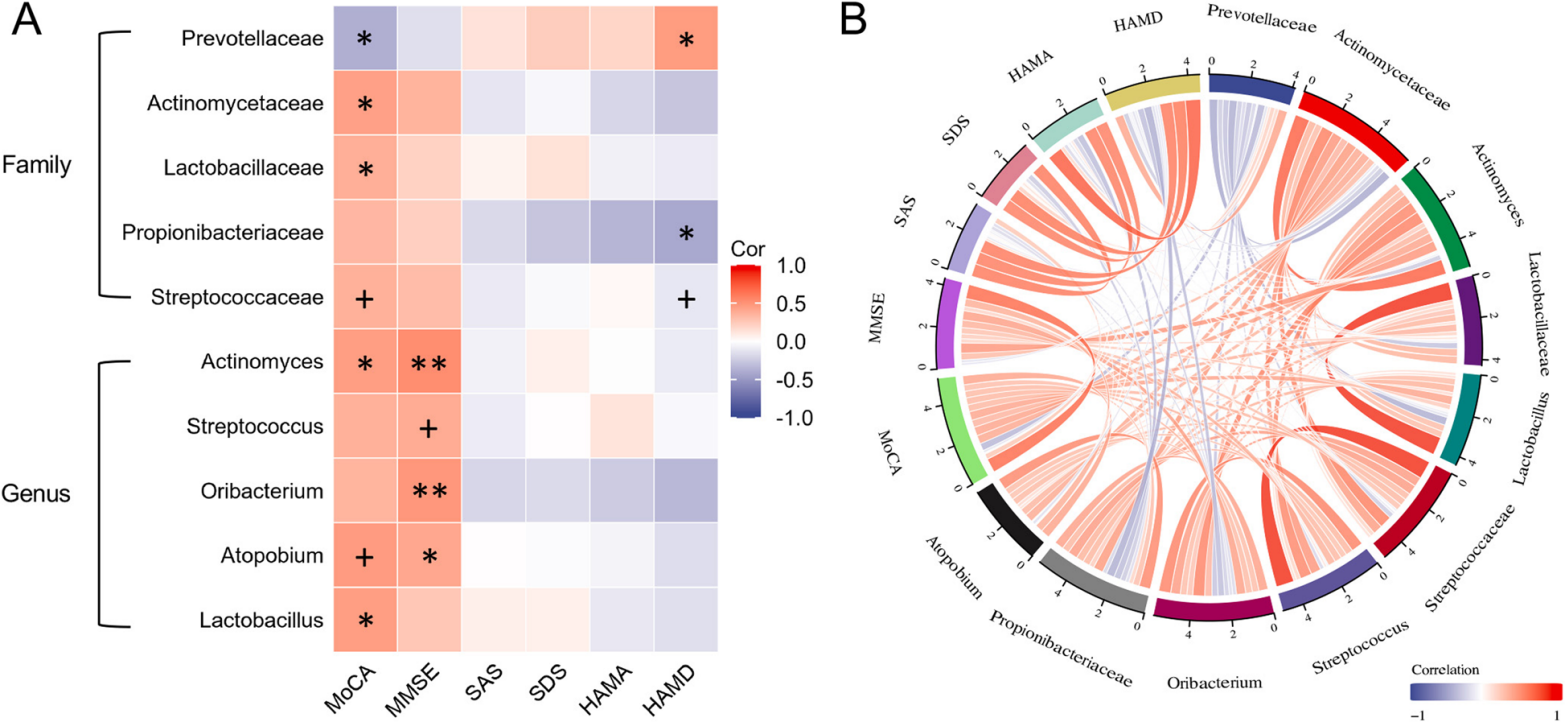
Correlation between gut microbiome and cognitive function.Correlation analysis with cognitive and emotional scores: (**A)** heatmap and **(B)** chordal graph. *P < 0.05, **P < 0.01, ^+^*age-adjust P* < 0.05. The correlation in both graphs is an unadjusted correlation, the age-adjust correlation is provided in Table S4. MoCA, Montreal cognitive assessment scale; MMSE, Mini-mental state examination; SAS, Self-rating anxiety scale; SDS, Self-rating depression scale; HAMA, Hamilton anxiety scale; HAMD, Hamilton depression scale

 A comparison of the clinical data between PNCI and PCI is summarized in Table 3 and Table S11. There were significant differences in age; years of education; CRP, C3, and C4 levels; and the MoCA and MMSE scores between the two groups (*P* < 0.05); however, there was no significant differences in gender, hypertension history, diabetes, and cardiovascular disease, total dialysate glucose content, and use of blood pressure medications (*P* > 0.05). At the family and genus levels, five gut microbiota, including *Actinomycetaceae*, *Lactobacillaceae*, *Actinomyces*, *Atopobium*, and *Lactobacillus*, significantly and positively correlated with the total MoCA score (*P* < 0.05), and one gut microbiota (*Prevotellaceae*) negatively correlated with it (*P* < 0.05). Moreover, four microbiota (*Actinomyces*, *Streptococcus*, *Oribacterium*, and *Atopobium*) positively correlated with the MMSE score (*P* < 0.05). In addition, microbiome alteration was associated with depression; the HAMD scores positively correlated with *Prevotellaceae* (*P* < 0.05) and negatively with *Propionibacteriaceae* (*P* < 0.05) (Fig. 5). Considering that the age difference can be a confounder factor, we recalculated the age-adjusted correlation using partial correlation analysis (Table S6). The results suggest that *Streptococcaceae* and *Atopobium* are still strongly correlated with MoCA scores after controlling for age (*P* < 0.05).

**Table 3:** Comparison of clinical data of subgroup

Variables	PNCI (*n* = 9)	PCI (*n* = 19)	t (Z) value	*P* value
**Sex (M/F)**	4 / 5	9 / 10	-0.142	0.887
**Age (Years)**	36.11 ± 11.80	50.32 ± 7.41	-3.905	0.001
**Education (Years)**	8.00 (8.00, 8.50)	6.00 (3.00, 8.00)	2.335	0.020
**BMI (kg/m** ^**2**^ **)**	20.42 ± 3.61	22.48 ± 2.42	-1.791	0.085
**Hypertension (yes/no)**	7 / 2	15 / 3	-0.069	0.945
**Diabetes (yes/no)**	1 / 8	2 / 17	-0.046	0.963
**Cardiovascular disease (yes/no)**	5 / 4	11 / 8	-0.378	0.706
**Total dialysate glucose content (g/d)**	4.50 (4.50, 6.50)	4.50 (7.00, 8.00)	-1.409	0.159
**ARB (yes/no)**	1 / 8	3 / 15	-0.324	0.746
**CCB (yes/no)**	6 / 3	14 / 5	-0.377	0.706
**β-receptor blocker (yes/no)**	2 / 7	7 / 12	-0.760	0.447
**TNF-a (pg/ml)**	19.50 (18.25, 22.45)	18.80 (16.50, 23.70)	-0.517	0.605
**IL-1β (pg/ml)**	5.29 (5.00, 21.45)	5.00 (5.00, 6.54)	-1.262	0.207
**CRP (mg/L)**	0.75 (0.60, 1.08)	1.81 (0.94, 8.97)	-2.533	0.011
WBC (*10 ^**9**^ **/L)**	6.21 ± 1.78	7.15 ± 2.26	-1.091	0.285
**Scr (mmol/L)**	1072.21 ± 295.01	1149.19 ± 264.01	-0.695	0.494
**Alb (g/L)**	38.92 ± 3.37	36.24 ± 3.77	1.817	0.081
**ALT (U/L)**	41.44 ± 55.66	23.47 ± 33.05	1.074	0.293
**AST(U/L)**	20.00 (15.50, 25.00)	18.00 (15.00, 20.00)	-1.087	0.277
**TG (mmol/L)**	1.22 (1.07, 2.09)	1.62 (0.87, 3.23)	-0.369	0.712
**TC (mmol/L)**	3.97 (3.36, 6.27)	4.37 (3.76, 5.38)	-0.344	0.731
**HDL (mmol/L)**	0.98 ± 0.21	1.06 ± 0.26	-0.776	0.445
**LDL (mmol/L)**	3.04 ± 1.45	2.85 ± 0.93	0.419	0.679
**C3 (g/L)**	0.84 ± 0.20	1.00 ± 0.11	-2.813	0.009
**C4 (g/L)**	0.27 ± 0.10	0.38 ± 0.09	-2.738	0.011
**PNI**	45.32 ± 5.04	42.29 ± 4.56	1.588	0.124
**MoCA**	27.33 ± 1.22	20.11 ± 3.98	7.220	0.000
**MMSE**	27.50 (27.00, 30.00)	26.00 (23.00, 28.00)	-2.757	0.006
**SAS**	35.56 ± 5.90	35.11 ± 7.29	0.201	0.843
**SDS**	37.11 ± 7.56	36.89 ± 9.20	0.061	0.952
**HAMA**	6.89 ± 3.89	9.32 ± 5.37	-1.210	0.237
**HAMD**	4.56 ± 2.96	6.47 ± 3.76	-1.341	0.192
**GSRS**	26.00 (20.00, 27.50)	22.00 (21.00, 26.00)	-0.322	0.747

Notes: PNI = Alb (g/L) + 5* [total lymphocyte count (*10^9^/L)]; “ARB, CCB and β-receptor blocker” represent the drug used in the previous month. The details of the patients are provided in Table S11

Abbreviations: *PNCI *Peritoneal dialysis patient with normal cognition, *PCI *Peritoneal dialysis patient with cognitive impairment, *ARB *Angiotensin receptor blocker, *CCB *Calcium channel blockers, *BMI *Body mass index, *TNF-α *Tumor necrosis factor-alpha, *IL-1β *Interleukin-1beta, *WBC *White blood cell, *CRP *C-reactive protein, *Scr *Serum creatinine, *Alb *Albumin, *ALT *Alanine aminotransferase, *AST *Aspartate aminotransferase, *TC *Total cholesterol, *TG *Triglyceride, *HDL *High-density lipoprotein, *LDL *Low- density lipoprotein, *C3 *Complement-3, *C4 *Complement-4, *PNI *Prognostic nutritional index, *MoCA *Montreal cognitive assessment scale, *MMSE *Mini-mental state examination, *SAS *Self-rating anxiety scale, *SDS *Self-rating depression scale, *HAMA *Hamilton anxiety scale, *HAMD *Hamilton depression scale, *GSRS *Gastrointestinal symptom rating scale

#### Receiver operating characteristic (ROC) analysis

**Fig. 6:**
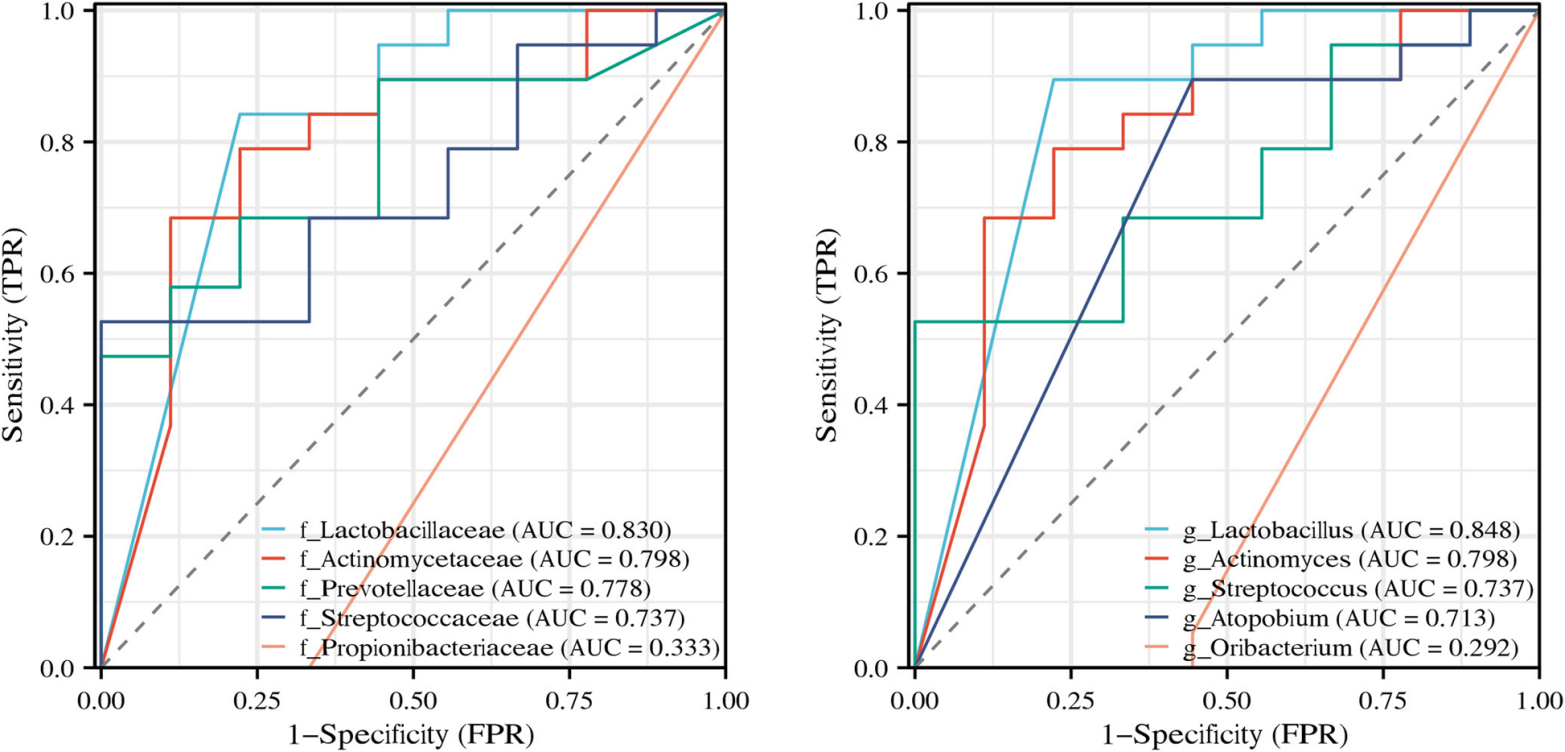
ROC analysis.f, family; g, genus

The MoCA score was used as the grouping standard to establish ROC curves of the differential bacteria. The higher the AUC of an ROC curve, the better the model is for distinguishing between binary classes. *Lactobacillaceae* (AUC = 0.83), *Actinomycetaceae* (AUC = 0.789), *Prevotellaceae* (AUC = 0.778), and *Streptococcaceae* (AUC = 0.737) at the family level and *Lactobacillus* (AUC = 0.848), *Actinomyces* (AUC = 0.798), *Streptococcus* (AUC = 0.737), and *Atopobium* (AUC = 0.713) at the genus level showed good distinguishing values in PCI (Table 4; Fig. 6).

**Table 4:** ROC curve analysis

Variables	AUC	95% CI	Cut-off	Sensitivity	Specificity	Youden index
**f_Lactobacillaceae**	0.830	0.661–1.000	0.001	0.842	0.778	0.620
**f_Actinomycetaceae**	0.798	0.605–0.991	0.003	0.684	0.889	0.573
**f_Prevotellaceae**	0.778	0.603–0.953	0.071	0.474	1.000	0.474
**f_Streptococcaceae**	0.737	0.548–0.926	0.007	0.526	1.000	0.526
**f_Propionibacteriaceae**	0.333	0.170–0.497	-Inf	1.000	0.000	0.000
**g_Lactobacillus**	0.848	0.687–1.000	0.001	0.895	0.778	0.673
**g_Actinomyces**	0.798	0.605–0.991	0.003	0.684	0.889	0.573
**g_Streptococcus**	0.737	0.548–0.926	0.007	0.526	1.000	0.526
**g_Atopobium**	0.713	0.526–0.901	0.001	0.895	0.556	0.450
**g_Oribacterium**	0.292	0.109–0.476	-Inf	1.000	0.000	0.000

Abbreviations: *AUC *Area under curve, *CI *Confidence interval, *f *family, *g *genus, *Inf *Infinity small

#### Functional difference analysis of the gut Microbiome

**Fig. 7:**
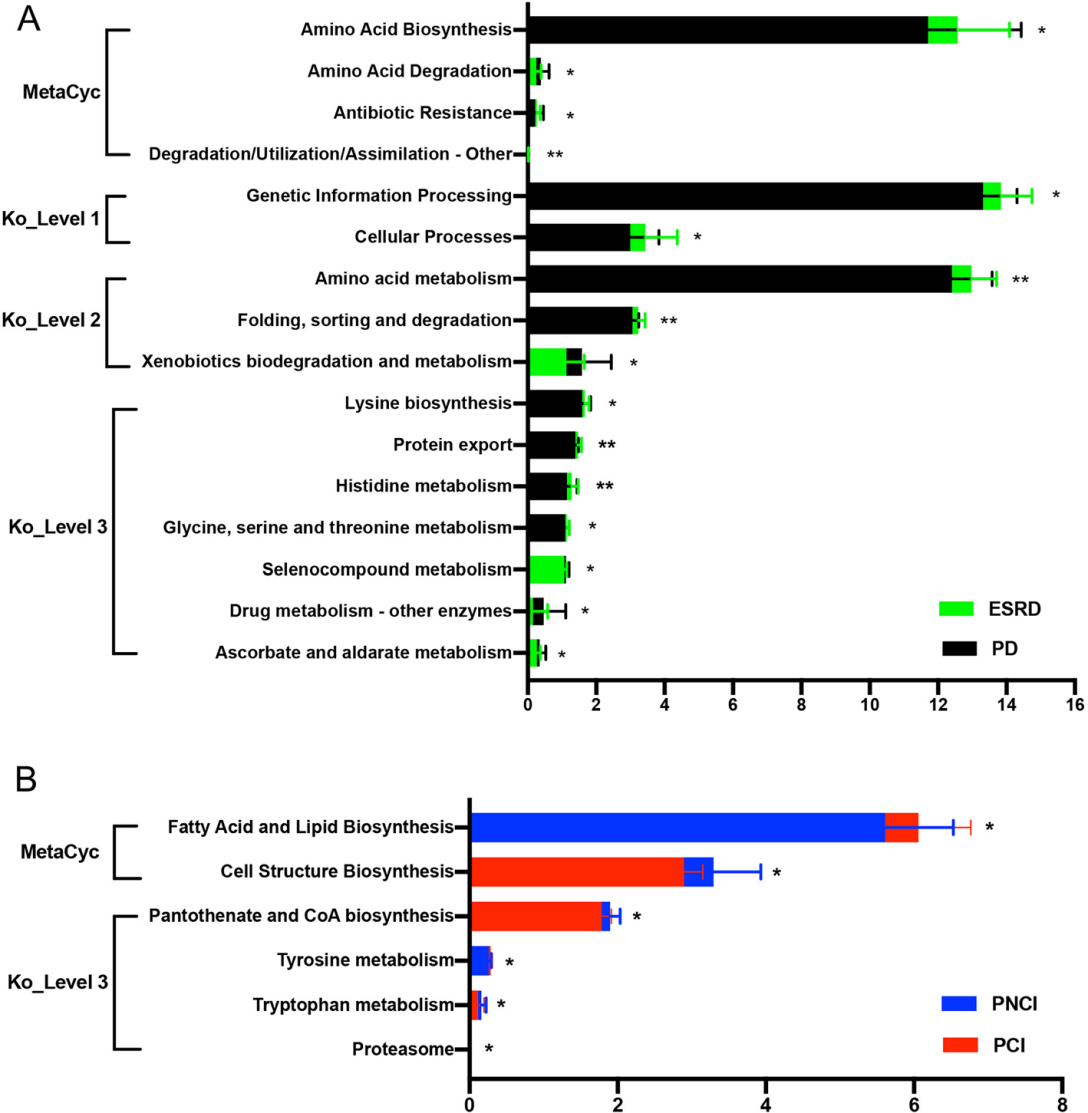
Function prediction. **A** Functional difference analysis between the non-dialysis ESRD and PD groups. **B** Functional difference analysis between the PNCI and PCI groups. *P < 0.05, **P < 0.01. ESRD, end-stage renal disease; PD, peritoneal dialysis; PCI, peritoneal dialysis patients with cognitive impairment; PNCI, peritoneal dialysis patients with normal cognition

The PICRUSt website was used to infer the function of the gut microbiome, and MetaCyc and KEGG data were used to enrich the metabolic pathways (Table 5, Table S7, S8, S9, Fig. 7). The function prediction results showed many different modules, such as seleno-compound metabolism, drug metabolism, and amino acid degradation and synthesis, which were differentially enriched between PD and non-dialysis ESRD. The enriched modules in PCI included fatty acid and lipid biosynthesis (second level of MetaCyc) and tyrosine metabolism (third level of KEGG). In contrast, cell structure biosynthesis (second level of MetaCyc), pantothenate and coenzyme A synthesis (third level of KEGG), tryptophan metabolism (third level of KEGG), and proteasomes (third level of KEGG) were decreased in PCI.

**Table 5:** Functional differences analysis

Subgroup	Methods	Change	Pathways	*P* value
**ESRD vs. PD**	MetaCyc	Up	Amino Acid Degradation	0.017
		Down	Amino Acid Biosynthesis	0.020
			Antibiotic Resistance	0.029
			Degradation/Utilization/Assimilation – Other	0.008
	Ko_Level 1	Down	Genetic Information Processing	0.032
			Cellular Processes	0.049
	Ko_Level 2	Up	Xenobiotics biodegradation and metabolism	0.017
		Down	Amino acid metabolism	0.002
			Folding, sorting and degradation	0.005
	Ko_Level 3	Up	Selenocompound metabolism	0.025
			Drug metabolism - other enzymes	0.036
			Ascorbate and aldarate metabolism	0.015
		Down	Lysine biosynthesis	0.024
			Protein export	0.002
			Histidine metabolism	0.002
			Glycine, serine and threonine metabolism	0.043
**PNCI vs. PCI**	MetaCyc	Up	Fatty Acid and Lipid Biosynthesis	0.049
		Down	Cell Structure Biosynthesis	0.044
	Ko_Level 3	Up	Tyrosine metabolism	0.044
		Down	Pantothenate and CoA biosynthesis	0.049
			Tryptophan metabolism	0.026
			Proteasome	0.027

Notes: “Up” represents a significant increase in the PD (or PCI) group (P < 0.05); “Down” represents significant decrease in the PD (or PCI) group (P < 0.05). The details of the results are shown in Table S7, S8, and S9

Abbreviations: *ESRD *End stage renal disease, *PD *Peritoneal dialysis, *PNCI *Peritoneal dialysis patient with normal cognition, *PCI *Peritoneal dialysis patient with cognitive impairment, *Ko *KEGG Ortholog

## Discussion

Kidney disease [9] and haemodialysis [29] can disrupt the gut microbiome. As a peritoneal cavity-dependent treatment, PD is closely related to peritoneal function and the gut microbiome. Currently, the gut microbiome in patients undergoing PD is believed to be unbalanced [30]; however, the gut microbiome between patients undergoing PD and their household contacts are not significantly different [31]. Our study further confirmed the differences in gut microbiome abundance and structure between patients undergoing PD and those with non-dialysis ESRD. In addition, the cognitive function score of the PD group was lower than that of the non-dialysis ESRD group, whereas the gastrointestinal symptom score was higher. The abundance and structure of the gut microbiome were also lower in PCI than in PNCI. Recently, Wang et al. demonstrated that gut microbiota alteration impaired the brain default mode network connectivity by enhancing systemic inflammation in patients with ESRD [9]. Additionally, gut microbiota and serum metabolites may be involved in the pathogenesis of haemodialysis-related mild cognitive decline [10]. However, the gut microbiome has not been reported to be involved in PD-related cognitive decline.

At the family and genus levels, the Wilcoxon test showed the most significant differences for *Lactobacillus*. LEfSe analysis suggested that *Prevotellaceae* had the highest grouping values, whereas correlation and ROC analyses indicated that multiple differential bacterial taxa had a significant relationship with serum index and cognitive function in patients undergoing PD. An increased abundance of *Prevotellaceae* has been observed in the guts of patients with schizophrenia [32] and cerebral palsy [33], and altered in the gut of a monkey model of major depression [34]. *Lactobacillus* plantarum treatment can regulate plasma trimethylamine oxide levels in APP/PS1 mice (an autosomal dominant mouse model), thereby improving cognitive status [35]. Our study showed that many differential bacterial taxa in the PD vs. non-dialysis ESRD groups were correlated with several clinical markers, including Alb, TC, and TG levels and the PNI. *Ruminococcaceae* is an important taxa, which plays a crucial role in the digestion of resistant starch and is associated with intestinal, immune, and nervous system diseases [29]. It has been reported to be linked to the severity of chronic kidney diseases, such as diabetic nephropathy [36] and idiopathic nephrotic syndrome [37]. Previous randomized controlled clinical trials have shown that oral administration of bacteria, such as *Bifidobacterium* and *Lactobacillus*, can help preserve residual kidney function in patients requiring PD [38]. Regulation of the intestinal microbiota through personalized diet and oral bacterial therapy can provide a new treatment plan for patients undergoing PD.

Clinical correlation results suggested that *Lactobacillaceae*, *Lactobacillus*, *Oribacterium*, and *Prevotellaceae* were associated with serum complement and CRP levels. Serum complement is an essential factor involved in the microbial defence response and immune regulation of the human body. Zhang et al.‘s study confirmed that complement plays a critical role in brain white matter damage [39], which is closely related to cognitive decline. Serum complement has also been associated with synapse loss in Alzheimer’s disease [40]. CRP is a commonly used clinical monitoring index and sensitive marker of non-specific inflammatory responses. High levels of CRP can increase the production of adhesion molecules and chemokines, regulate monocyte accumulation, and promote vascular inflammation [41]. Relevant data have shown that CRP levels are associated with dementia [42] and schizophrenia [43].

The function prediction results showed differences in modules related to fatty acid, lipid, pantothenate, and coenzyme A biosynthesis, and tyrosine and tryptophan metabolism between PCI and PNCI. Previous prospective follow-up studies have suggested that adequate pantothenate supplementation could help prevent cognitive decline in older patients with diabetes [44]. Short-chain fatty acids (SCFAs) are metabolites of the intestinal microorganisms. SCFAs can regulate neurotrophic factors and neuroinflammation by affecting the morphology and function of microglia [45]; thus, they have been widely reported to be related to cognitive state [46]. The potential modulation of intestinal amino acids metabolism is an important factor in affecting the gut-brain axis. Consistent with previous evidence, Liu et al. also detected changes in tryptophan and tyrosine metabolism using faecal sequencing in Alzheimer’s disease [7]. Accumulating evidence reveals that the gut microbiota has versatile impacts on intestinal tryptophan, including tryptophan degradation [47], the serotonin synthesis pathway [48], and kynurenine pathway [49]. Tryptophan is the sole precursor of serotonin, which is an important monoamine neurotransmitter in central nervous system development [48]. Additionally, the activation of kynurenine pathway has been linked to a reduced hippocampal volume, resulting in memory loss in those with severe depression [50]. Furthermore, tyrosine levels are associated with oxidative stress in the brain and astrocytes, leading to cognitive impairment [51]. Sarkis et al. found that gut microbiota can metabolize tyrosine into 4EP, which is then converted into 4EPS under the action of host sulfotransferase (SULT1A1). 4EPS can enter the brain of mice, affect the activation and connections of specific brain areas, and regulate the brain activity and anxiety-like behaviours of mice [52]. Taken together, these functions inferred by PICRUSt present new opportunities for future research on PD-related cognitive impairment.

**Fig. 8:**
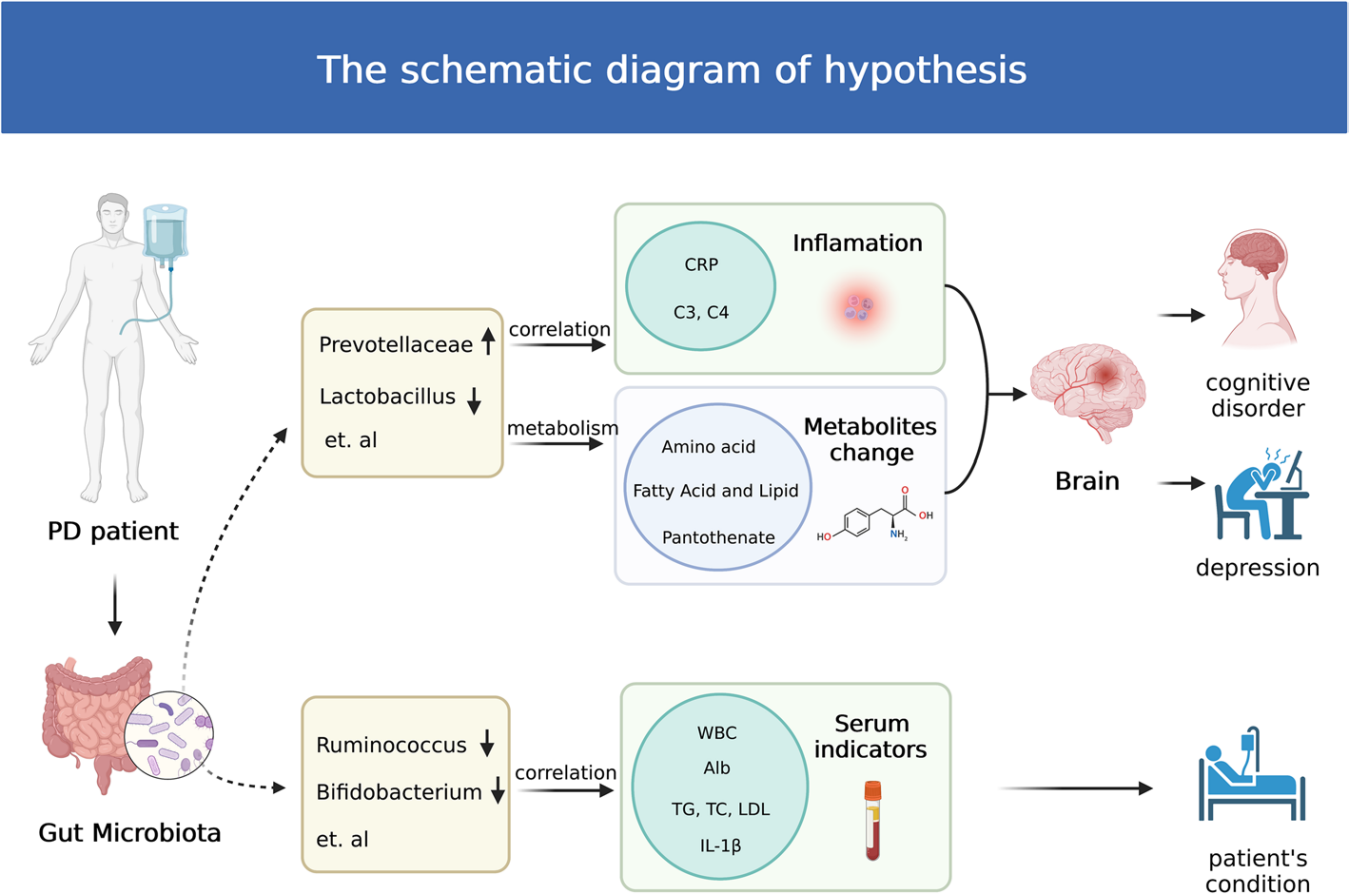
The schematic of the hypothesis. PD, peritoneal dialysis; WBC, white blood cell; interleukin-1beta, IL-1β; CRP, C-reactive protein; Alb, albumin; TC, total cholesterol; TG, triglyceride; HDL, high-density lipoprotein; LDL, low-density lipoprotein; C3, complement-3; C4, complement-4

In summary, we propose the following hypothesis (Fig. 8): Patients undergoing PD have gut microbiome disorders. The bacterial communities, represented by *Prevotellaceae* and *Lactobacillus*, differed in terms of the metabolic functions of amino acids, fatty acids, and pantothenate and were correlated with serum indices, such as CRP, C3, and C4 levels, thereby affecting the cognitive function and depressive mood of patients. The different bacterial communities represented by *Ruminococcaceae* and *Bifidobacteriaceae* are associated with serum indices, such as WBC counts and Alb, TG, TC, LDL, and IL-1β levels, and are thereby associated with the disease status of patients. Evaluating the gut microbiome of patients undergoing PD could help monitor their cognitive and disease statuses, effectively preventing and delaying the occurrence of cognitive disorders.

This study had some limitations. First, the sample size was small. Second, routine follow-up was not performed. Therefore, any association between the differential microbiota and prognosis of patients undergoing PD remains unclear. Third, the information gained from the PICRUSt website is only inferred function, which has not been measured experimentally or clinically; therefore, these results need to be further explored and validated in future studies.

## Conclusion

This study is the first to describe the differences in faecal microbiota between patients undergoing PD with and without cognitive impairment. Our findings demonstrate that the faecal microbial composition of PCI undergoing PD is altered, characterized by abnormal microbiota function related to amino acid and lipid metabolisms. The bacterial community represented by *Prevotellaceae* and *Lactobacillus* is correlated with cognitive scores and has good value in the differential diagnosis of PCI. The knowledge gained from this study will facilitate early diagnosis and therapeutic attempts to target the commensal microbiota in patients undergoing PD with cognitive impairment.

### Supplementary Information

**Additional file 1: Figure S1.** Cumulative curve.

**Additional file 2: Table S1.** Comparison of alpha and beta diversity.

**Additional file 3: Table S2.** Proportion of gut microbiome in the phylum classification.

**Additional file 4: Table S3.** Comparison results of gut microbiomes with differences in abundance between PD and ESRD.

**Additional file 5: Table S4.** Comparison results of gut microbiomes with differences in abundance between PCI and PNCI.

**Additional file 6: Table S5.** Significant correlation between gut microbiota and clinical markers.

**Additional file 7: Table S6.** The calculation results (unadjusted, and age-adjusted) of the correlation between gut microbiota and cognitive function.

**Additional file 8: Table S7.** The KEGG analysis between PD and ESRD.

**Additional file 9: Table S8.** The KEGG analysis between PCI and PNCI.

**Additional file 10: Table S9.** The MetaCys analysis results.

**Additional file 11: Table S10.** The clinical detail of each patient in PD and ESRD groups.

**Additional file 12: Table S11.** The clinical detail of each patient in PCI and PNCI groups.

## Data Availability

SRA records of metagenomic data from this work are available via the following link: https://www.ncbi.nlm.nih.gov/sra/PRJNA925078. Further inquiries can be directed to the corresponding author.

## Abbreviations

PD: Peritoneal dialysis
ESRD: End-stage renal disease
PCI: PD patients with cognitive impairment
PNCI: PD patients with normal cognition
MoCA: Montreal Cognitive Assessment
MMSE: Mini-Mental State Examination
SDS: Self-rating depression scale
SAS: Self-rating anxiety scale
HAMA: Hamilton anxiety scale
HAMD: Hamilton depression scale
GSRS: Gastrointestinal symptom rating scale
16S RNA: 16S ribosomal ribonucleic acid
PCoA: Principal coordinate analysis
ANOSIM: Analysis of similarities
FDR: False discovery rate
KEGG: Kyoto Encyclopedia of Genes and Genomes
LDA: Linear discriminant analysis
AUC: Area under the curve
IL-1β: Interleukin-1 beta
CRP: C-reactive protein
SCFAs: Short-chain fatty acids

## References

[1] Tangwonglert T, Davenport A (2020). Differences in predicting glucose absorption from peritoneal dialysate compared to measured absorption in peritoneal dialysis patients treated by continuous ambulatory peritoneal dialysis and ambulatory peritoneal dialysis cyclers. Int J Artif Organs.

[2] Liao JL, Zhang YH, Xiong ZB, Hao L, Liu GL, Ren YP (2019). The Association of cognitive impairment with peritoneal dialysis-related Peritonitis. Perit Dial Int.

[3] Griva K, Stygall J, Hankins M, Davenport A, Harrison M, Newman SP (2010). Cognitive impairment and 7-year mortality in dialysis patients. Am J Kidney Dis.

[4] Smith PA (2015). The tantalizing links between gut microbes and the brain. Nature.

[5] Mayer EA, Knight R, Mazmanian SK, Cryan JF, Tillisch K (2014). Gut microbes and the brain: paradigm shift in neuroscience. J Neurosci.

[6] Yang T, Richards EM, Pepine CJ, Raizada MK (2018). The gut microbiota and the brain-gut-kidney axis in Hypertension and chronic Kidney Disease. Nat Rev Nephrol.

[7] Liu P, Wu L, Peng G, Han Y, Tang R, Ge J (2019). Altered microbiomes distinguish Alzheimer’s Disease from amnestic mild cognitive impairment and health in a Chinese cohort. Brain Behav Immun.

[8] Qu L, Dong Z, Ma S, Liu Y, Zhou W, Wang Z (2022). Gut microbiome signatures are predictive of cognitive impairment in Hypertension patients-a cohort study. Front Microbiol.

[9] Wang YF, Zheng LJ, Liu Y, Ye YB, Luo S, Lu GM (2019). The gut microbiota-inflammation-brain axis in end-stage renal Disease: perspectives from default mode network. Theranostics.

[10] Zhu B, Shen J, Jiang R, Jin L, Zhan G, Liu J (2020). Abnormalities in gut microbiota and serum metabolites in hemodialysis patients with mild cognitive decline: a single-center observational study. Psychopharmacology.

[11] Bennett PN, Bohm C, Harasemiw O, Brown L, Gabrys I, Jegatheesan D (2022). Physical activity and exercise in peritoneal dialysis: International Society for Peritoneal Dialysis and the Global Renal Exercise Network practice recommendations. Perit Dial Int.

[12] Teitelbaum I, Glickman J, Neu A, Neumann J, Rivara MB, Shen J (2021). KDOQI US commentary on the 2020 ISPD practice recommendations for prescribing high-quality goal-directed peritoneal dialysis. Am J Kidney Dis.

[13] Shlipak MG, Tummalapalli SL, Boulware LE, Grams ME, Ix JH, Jha V (2021). The case for early identification and intervention of chronic kidney disease: conclusions from a Kidney Disease: Improving Global Outcomes (KDIGO) Controversies Conference. Kidney Int.

[14] Ikizler TA, Burrowes JD, Byham-Gray LD, Campbell KL, Carrero JJ, Chan W (2020). KDOQI clinical practice guideline for nutrition in CKD: 2020 update. Am J Kidney Dis.

[15] Katzman R, Zhang MY, Ouang Ya Q, Wang ZY, Liu WT, Yu E (1988). A Chinese version of the Mini-mental State examination; impact of illiteracy in a Shanghai Dementia survey. J Clin Epidemiol.

[16] Nasreddine ZS, Phillips NA, Bedirian V, Charbonneau S, Whitehead V, Collin I (2005). The Montreal Cognitive Assessment, MoCA: a brief screening tool for mild cognitive impairment. J Am Geriatr Soc.

[17] Faravelli C, Albanesi G, Poli E (1986). Assessment of depression: a comparison of rating scales. J Affect Disord.

[18] Hamilton M (1960). A rating scale for depression. J Neurol Neurosurg Psychiatry.

[19] Zung WW (1971). A rating instrument for anxiety disorders. Psychosomatics.

[20] Hamilton M (1959). The assessment of anxiety states by rating. Br J Med Psychol.

[21] Svedlund J, Sjodin I, Dotevall G (1988). GSRS–a clinical rating scale for gastrointestinal symptoms in patients with irritable bowel syndrome and Peptic Ulcer Disease. Dig Dis Sci.

[22] Jia X, Wang Z, Huang F, Su C, Du W, Jiang H (2021). A comparison of the Mini-mental State Examination (MMSE) with the Montreal Cognitive Assessment (MoCA) for mild cognitive impairment screening in Chinese middle-aged and older population: a cross-sectional study. BMC Psychiatry.

[23] Yu J, Li J, Huang X (2012). The Beijing version of the Montreal Cognitive Assessment as a brief screening tool for mild cognitive impairment: a community-based study. BMC Psychiatry.

[24] Wang J, Wang X, Wang M, Wang J, Wu Y, Qi X (2023). Clinical significance of Interleukin 17 receptor E in diabetic Nephropathy. Int Immunopharmacol.

[25] Chong J, Liu P, Zhou G, Xia J (2020). Using MicrobiomeAnalyst for comprehensive statistical, functional, and meta-analysis of microbiome data. Nat Protoc.

[26] Wilkinson TJ, Huws SA, Edwards JE, Kingston-Smith AH, Siu-Ting K, Hughes M (2018). CowPI: a rumen microbiome focussed version of the PICRUSt functional inference software. Front Microbiol.

[27] Genovese CR, Lazar NA, Nichols T (2002). Thresholding of statistical maps in functional neuroimaging using the false discovery rate. Neuroimage.

[28] Onodera T, Goseki N, Kosaki G (1984). [Prognostic nutritional index in gastrointestinal Surgery of malnourished cancer patients]. Nihon Geka Gakkai Zasshi.

[29] Bloemendaal M, Szopinska-Tokov J, Belzer C, Boverhoff D, Papalini S, Michels F (2021). Probiotics-induced changes in gut microbial composition and its effects on cognitive performance after stress: exploratory analyses. Transl Psychiatry.

[30] Merino-Ribas A, Araujo R, Pereira L, Campos J, Barreiros L, Segundo MA (2022). Vascular calcification and the gut and blood microbiome in chronic Kidney Disease patients on peritoneal dialysis: a pilot study. Biomolecules.

[31] Teixeira RR, de Andrade LS, Pereira NBF, Montenegro H, Hoffmann C, Cuppari L (2023). Gut microbiota profile of patients on peritoneal dialysis: comparison with household contacts. Eur J Clin Nutr.

[32] Ni JJ, Xu Q, Yan SS, Han BX, Zhang H, Wei XT (2021). Gut microbiota and psychiatric disorders: a two-sample mendelian randomization study. Front Microbiol.

[33] Huang C, Li Y, Feng X, Li D, Li X, Ouyang Q (2019). Distinct gut microbiota composition and functional category in children with cerebral palsy and Epilepsy. Front Pediatr.

[34] Teng T, Clarke G, Maes M, Jiang Y, Wang J, Li X (2022). Biogeography of the large intestinal mucosal and luminal microbiome in cynomolgus macaques with depressive-like behavior. Mol Psychiatry.

[35] Wang QJ, Shen YE, Wang X, Fu S, Zhang X, Zhang YN (2020). Concomitant memantine and Lactobacillus plantarum treatment attenuates cognitive impairments in APP/PS1 mice. Aging.

[36] Chen R, Zhu D, Yang R, Wu Z, Xu N, Chen F (2022). Gut microbiota diversity in middle-aged and elderly patients with end-stage diabetic Kidney Disease. Ann Transl Med.

[37] He H, Lin M, You L, Chen T, Liang Z, Li D (2021). Gut microbiota profile in adult patients with idiopathic Nephrotic Syndrome. Biomed Res Int.

[38] Wang IK, Wu YY, Yang YF, Ting IW, Lin CC, Yen TH (2015). The effect of probiotics on serum levels of cytokine and endotoxin in peritoneal dialysis patients: a randomised, double-blind, placebo-controlled trial. Benef Microbes.

[39] Zhang LY, Pan J, Mamtilahun M, Zhu Y, Wang L, Venkatesh A (2020). Microglia exacerbate white matter injury via complement C3/C3aR pathway after hypoperfusion. Theranostics.

[40] Hong S, Beja-Glasser VF, Nfonoyim BM, Frouin A, Li S, Ramakrishnan S (2016). Complement and microglia mediate early synapse loss in Alzheimer mouse models. Science.

[41] Gao CZ, Ma QQ, Wu J, Liu R, Wang F, Bai J (2018). Comparison of the effects of ticagrelor and clopidogrel on inflammatory factors, vascular endothelium functions and short-term prognosis in patients with acute ST-segment elevation Myocardial Infarction undergoing emergency percutaneous coronary intervention: a pilot study. Cell Physiol Biochem.

[42] Ng A, Tam WW, Zhang MW, Ho CS, Husain SF, McIntyre RS (2018). IL-1beta, IL-6, TNF- alpha and CRP in elderly patients with depression or Alzheimer’s Disease: systematic review and Meta-analysis. Sci Rep.

[43] Bora E (2019). Peripheral inflammatory and neurotrophic biomarkers of cognitive impairment in schizophrenia: a meta-analysis. Psychol Med.

[44] Araki A, Yoshimura Y, Sakurai T, Umegaki H, Kamada C, Iimuro S (2017). Low intakes of carotene, vitamin B(2), pantothenate and calcium predict cognitive decline among elderly patients with Diabetes Mellitus: the Japanese Elderly Diabetes intervention trial. Geriatr Gerontol Int.

[45] Zhou Z, Xu N, Matei N, McBride DW, Ding Y, Liang H (2021). Sodium butyrate attenuated neuronal apoptosis via GPR41/Gbetagamma/PI3K/Akt pathway after MCAO in rats. J Cereb Blood Flow Metab.

[46] Lee J, Venna VR, Durgan DJ, Shi H, Hudobenko J, Putluri N (2020). Young versus aged microbiota transplants to germ-free mice: increased short-chain fatty acids and improved cognitive performance. Gut Microbes.

[47] Kaluzna-Czaplinska J, Gatarek P, Chirumbolo S, Chartrand MS, Bjorklund G (2019). How important is tryptophan in human health?. Crit Rev Food Sci Nutr.

[48] O’Mahony SM, Clarke G, Borre YE, Dinan TG, Cryan JF (2015). Serotonin, tryptophan metabolism and the brain-gut-microbiome axis. Behav Brain Res.

[49] Desbonnet L, Clarke G, Traplin A, O’Sullivan O, Crispie F, Moloney RD (2015). Gut microbiota depletion from early adolescence in mice: implications for brain and behaviour. Brain Behav Immun.

[50] Peukert X, Steindorf K, Schagen SB, Runz A, Meyer P, Zimmer P (2020). Hippocampus-related cognitive and affective impairments in patients with breast cancer-a systematic review. Front Oncol.

[51] de Oliveira J, Farias HR, Streck EL (2021). Experimental evidence of tyrosine neurotoxicity: focus on mitochondrial dysfunction. Metab Brain Dis.

[52] Needham BD, Funabashi M, Adame MD, Wang Z, Boktor JC, Haney J (2022). A gut-derived metabolite alters brain activity and anxiety behaviour in mice. Nature.

