# Supplementary figures and images for: Correlation between gut microbiome and cognitive impairment in patients undergoing peritoneal dialysis
Source: BMC Nephrol. 2023 Dec 5;24:360. doi: 10.1186/s12882-023-03410-z (PMC10696889; doi:10.1186/s12882-023-03410-z)

**Figure S1.** Cumulative curve.

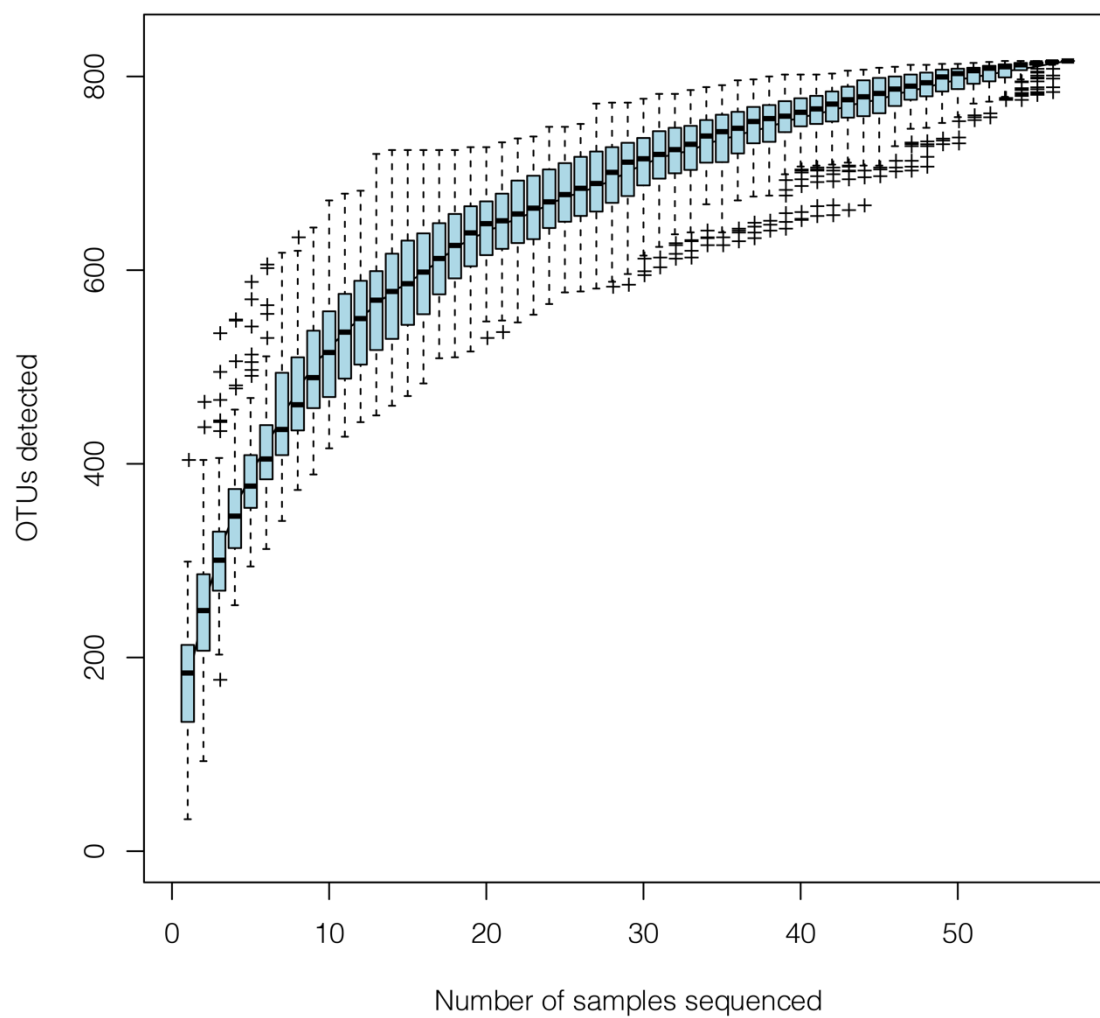

Supplement: Supplementary file 1 — Additional file 1: Figure S1. Cumulative curve. [file 12882_2023_3410_MOESM1_ESM.pdf]
