# Supplementary material for: Correlation between gut microbiome and cognitive impairment in patients undergoing peritoneal dialysis
Source: BMC Nephrol. 2023 Dec 5;24:360. doi: 10.1186/s12882-023-03410-z (PMC10696889; doi:10.1186/s12882-023-03410-z)
Supplement: Supplementary file 2 — Additional file 2: Table S1. Comparison of alpha and beta diversity. [file 12882_2023_3410_MOESM2_ESM.pdf]

Table S1. Comparison of alpha and beta diversity.

Group1	Group2	alpha diversity		beta diversity			
		Simpson P value	Shannon P value	Axls.1	Axls.2	R	P value
ESRD	PD	0.012	0.008	13.9%	17.7%%	0.046	< 0.031
PNCI	PCI	0.048	0.128	15.2%	18.3%	0.202	< 0.025
NCI	CI	0.208	0.468	14.0%	22.1%	0.020	< 0.358

Abbreviations: ESRD, end stage renal disease; PD, peritoneal dialysis; PNCI, peritoneal dialysis patient with normal cognition; PCI, peritoneal dialysis patient with cognitive impairment.
