# Supplementary material for: Correlation between gut microbiome and cognitive impairment in patients undergoing peritoneal dialysis
Source: BMC Nephrol. 2023 Dec 5;24:360. doi: 10.1186/s12882-023-03410-z (PMC10696889; doi:10.1186/s12882-023-03410-z)
Supplement: Supplementary file 3 — Additional file 3: Table S2. Proportion of gut microbiome in the phylum classification. [file 12882_2023_3410_MOESM3_ESM.pdf]

Table S2. Proportion of gut microbiome in the phylum classification.

	ESRD		PD		PCI		PNCI	
Taxa	Abundance	Percentage	Abundance	Percentage	Abundance	Percentage	Abundance	Percentage
Bacteroidetes	550096	43.1	655431	47.77	449371	49.82	206060	43.84
Firmicutes	538038	42.16	481794	35.12	265952	29.48	215892	45.93
Proteobacteria	150763	11.81	205951	15.01	159859	17.72	46092	9.81
Actinobacteria	29333	2.3	3210	0.23	1566	0.17	1644	0.35
Fusobacteria	8026	0.63	25624	1.87	25296	2.8	328	0.07

Abbreviations: ESRD, end stage renal disease; PD, peritoneal dialysis; PNCI, peritoneal dialysis patient with normal cognition; PCI, peritoneal dialysis patient with cognitive impairment.
