# Supplementary material for: Correlation between gut microbiome and cognitive impairment in patients undergoing peritoneal dialysis
Source: BMC Nephrol. 2023 Dec 5;24:360. doi: 10.1186/s12882-023-03410-z (PMC10696889; doi:10.1186/s12882-023-03410-z)
Supplement: Supplementary file 4 — Additional file 4: Table S3. Comparison results of gut microbiomes with differences in abundance between PD and ESRD. [file 12882_2023_3410_MOESM4_ESM.pdf]

Table S3. Comparison results of gut microbiomes with differences in abundance between PD and ESRD.

Microbiota	Mean (CKD5)	SD (CKD5)	Mean (PD)	SD (PD)	P value
p_Actinobacteria	1.796	6.654	0.338	0.511	0.045
p_Firmicutes	46.725	25.178	35.172	24.540	0.047
p_Tenericutes	0.000	0.000	0.000	0.002	0.038
c_Actinobacteria	1.796	6.654	0.338	0.511	0.045
c_Clostridia	35.134	25.287	20.473	16.096	0.014
c_Mollicutes	0.000	0.000	0.000	0.002	0.038
o_Bifidobacteriales	1.451	6.648	0.111	0.302	0.050
o_Clostridiales	35.132	25.287	20.473	16.095	0.014
o_Mycoplasmatales	0.000	0.000	0.000	0.002	0.038
f_Bifidobacteriaceae	1.451	6.648	0.111	0.302	0.050
f_Christensenellaceae	0.000	0.000	0.002	0.003	0.044
f_Mycoplasmataceae	0.000	0.000	0.000	0.002	0.038
f_Peptoniphilaceae	0.000	0.000	0.002	0.006	0.010
f_Ruminococcaceae	13.558	11.623	8.242	8.955	0.027
g_Anaerosporobacter	0.049	0.121	0.001	0.006	0.006
g_Bifidobacterium	1.451	6.648	0.094	0.298	0.004
g_Butyricicoccus	0.085	0.100	0.054	0.094	0.013
g_Christensenella	0.000	0.000	0.002	0.003	0.044
g_Coprococcus	0.187	0.596	0.041	0.152	0.001
g_Dialister	0.371	1.100	0.765	3.360	0.048
g_Dorea	0.096	0.117	0.025	0.059	0.003
g_Fusicatenibacter	0.376	0.519	0.368	1.363	0.024
g_Gemmiger	0.835	1.672	0.156	0.612	0.000
g_Mycoplasma	0.000	0.000	0.000	0.002	0.038
g_Olsenella	0.000	0.000	0.005	0.023	0.011
g_Parasutterella	1.441	5.219	0.148	0.614	0.002
g_Peptoniphilus	0.000	0.000	0.002	0.006	0.010
g_Ruminococcus2	0.721	0.870	0.686	1.354	0.025
g_Terrisporobacter	0.037	0.172	0.001	0.004	0.007
p_Actinobacteria	1.796	6.654	0.338	0.511	0.045
p_Firmicutes	46.725	25.178	35.172	24.540	0.047
p_Tenericutes	0.000	0.000	0.000	0.002	0.038
s_Anaerosporobacter_mobilis	0.049	0.121	0.001	0.006	0.006
s_Anaerostipes_caccae	0.001	0.005	0.008	0.015	0.002
s_Anaerostipes_hadrus	0.106	0.155	0.036	0.067	0.024
s_Bacteroides_massiliensis	1.249	2.434	0.674	2.437	0.034
s_Bifidobacterium_pseudolongum	1.451	6.648	0.094	0.298	0.004
s_Blautia_luti	0.071	0.095	0.015	0.031	0.002
s_Blautia_obeum	0.039	0.080	0.025	0.059	0.016
s_Blautia_producta	0.007	0.015	0.001	0.005	0.023

s_Blautia_schinkii	0.012	0.020	0.004	0.008	0.049
s_Butyricicoccus_pullicaecorum	0.085	0.100	0.054	0.094	0.013
s_Christensenella_minuta	0.000	0.000	0.002	0.003	0.044
s_Clostridium_aldenense	0.038	0.074	0.099	0.133	0.015
s_Clostridium_fimetarium	0.136	0.564	0.015	0.079	0.014
s_Clostridium_methylpentosum	0.039	0.057	0.018	0.027	0.029
s_Clostridium_ramosum	0.046	0.084	0.104	0.216	0.019
s_Clostridium_scindens	0.004	0.010	0.013	0.019	0.023
s_Clostridium_tarantellae	0.005	0.011	0.000	0.001	0.001
s_Collinsella_aerofaciens	0.144	0.257	0.055	0.179	0.021
s_Coprococcus_catus	0.119	0.450	0.007	0.027	0.000
s_Dialister_invisus	0.320	1.095	0.760	3.361	0.024
s_Dorea_longicatena	0.096	0.117	0.025	0.059	0.003
s_Eubacterium_eligens	0.830	1.104	1.480	4.167	0.039
s_Faecalicoccus_acidiformans	0.023	0.063	0.004	0.014	0.005
s_Faecalicoccus_pleomorphus	0.013	0.020	0.048	0.087	0.014
s_Fusicatenibacter_saccharivorans	0.376	0.519	0.368	1.363	0.024
s_Gemmiger_formicilis	0.835	1.672	0.156	0.612	0.000
s_Mycoplasma_hominis	0.000	0.000	0.000	0.002	0.038
s_Parasutterella_excrementihominis	1.441	5.219	0.148	0.614	0.001
s_Prevotella_timonensis	0.000	0.000	0.006	0.021	0.017
s_Roseburia_inulinivorans	1.389	2.468	0.631	1.648	0.005
s_Ruminococcus_champanellensis	0.194	0.552	0.016	0.054	0.010
s_Ruminococcus_faecis	0.238	0.356	0.151	0.400	0.014
s_Ruminococcus_lactaris	0.078	0.169	0.001	0.003	0.000
s_Terrisporobacter_glycolicus	0.037	0.172	0.001	0.004	0.007

Abbreviations: ESRD, end stage renal disease; PD, peritoneal dialysis; p, phylum; c, class; o, order; f, family; g, genus; s, species.
