# Supplementary material for: Correlation between gut microbiome and cognitive impairment in patients undergoing peritoneal dialysis
Source: BMC Nephrol. 2023 Dec 5;24:360. doi: 10.1186/s12882-023-03410-z (PMC10696889; doi:10.1186/s12882-023-03410-z)
Supplement: Supplementary file 5 — Additional file 5: Table S4. Comparison results of gut microbiomes with differences in abundance between PCI and PNCI. [file 12882_2023_3410_MOESM5_ESM.pdf]

Table S4. Comparison results of gut microbiomes with differences in abundance between PCI and PNCI.

Microbiota	Mean (PCI)	SD (PCI)	Mean (PNCI)	SD (PNCI)	P value
o_Actinomycetales	0.007	0.009	0.066	0.124	0.037
f_Actinomycetaceae	0.005	0.008	0.049	0.106	0.012
f_Lactobacillaceae	0.000	0.001	0.013	0.024	0.001
f_Prevotellaceae	7.825	15.019	0.010	0.018	0.021
f_Propionibacteriaceae	0.000	0.000	0.002	0.005	0.010
f_Streptococcaceae	0.155	0.396	0.468	0.672	0.049
g_Actinomyces	0.005	0.008	0.049	0.106	0.012
g_Atopobium	0.000	0.001	0.002	0.002	0.020
g_Lactobacillus	0.000	0.001	0.013	0.024	0.000
g_Oribacterium	0.000	0.000	0.003	0.007	0.010
g_Streptococcus	0.154	0.396	0.468	0.672	0.049
s_Actinomyces_dentalis	0.000	0.000	0.001	0.002	0.043
s_Actinomyces_odontolyticus	0.004	0.007	0.046	0.104	0.013
s_Alistipes_indistinctus	0.032	0.070	0.004	0.013	0.044
s_Atopobium_rimae	0.000	0.001	0.001	0.002	0.022
s_Butyricimonas_virosa	0.116	0.306	0.000	0.000	0.029
s_Clostridium_butyricum	0.000	0.000	0.001	0.003	0.042
s_Clostridium_colinum	0.000	0.000	0.037	0.087	0.042
s_Lachnoanaerobaculum_umeaense	0.000	0.000	0.004	0.009	0.042
s_Lactobacillus_fermentum	0.000	0.000	0.003	0.009	0.042
s_Lactobacillus_iners	0.000	0.000	0.002	0.003	0.042
s_Oribacterium_sinus	0.000	0.000	0.003	0.007	0.010
s_Ruminococcus_champanellensis	0.003	0.010	0.044	0.092	0.010

Abbreviations: PNCI, peritoneal dialysis patient with normal cognition; PCI, peritoneal dialysis patient with cognitive impairment; o, order; f, family; g, genus; s, species.
