# Supplementary material for: Correlation between gut microbiome and cognitive impairment in patients undergoing peritoneal dialysis
Source: BMC Nephrol. 2023 Dec 5;24:360. doi: 10.1186/s12882-023-03410-z (PMC10696889; doi:10.1186/s12882-023-03410-z)
Supplement: Supplementary file 6 — Additional file 6: Table S5. Significant correlation between gut microbiota and clinical markers. [file 12882_2023_3410_MOESM6_ESM.pdf]

Table S5. Significant correlation between gut microbiota and clinical markers.

Factor 1	Factor 2	r	P value	FDR
IL-1β	g_Ruminococcus2	-0.406	0.032	0.1825
IL-1β	g_Parasutterella	-0.386	0.042	0.2401
CRP	f_Lactobacillaceae	-0.397	0.037	0.1777
CRP	g_Lactobacillus	-0.471	0.011	0.0645
WBC	f_Peptoniphilaceae	-0.418	0.027	0.2276
WBC	g_Olsenella	-0.596	0.001	0.0069
Alb	g_Bifidobacterium	0.510	0.006	0.0474
Alb	g_Anaerosporobacter	0.446	0.017	0.1472
ALT	g_Fusicatenibacter	0.472	0.011	0.0951
AST	g_Bifidobacterium	-0.482	0.009	0.0497
AST	g_Parasutterella	-0.392	0.039	0.2401
AST	g_Terrisporobacter	-0.396	0.037	0.3137
TG	g_Ruminococcus2	-0.414	0.029	0.1825
TG	g_Dorea	-0.405	0.032	0.2758
TC	g_Olsenella	0.440	0.019	0.0812
HDL	g_Peptoniphilus	0.424	0.025	0.2091
LDL	g_Olsenella	0.466	0.012	0.0700
C3	f_Lactobacillaceae	-0.387	0.042	0.1777
C3	g_Lactobacillus	-0.416	0.028	0.1183
C4	f_Lactobacillaceae	-0.458	0.014	0.1209
C4	g_Lactobacillus	-0.482	0.009	0.0645
C4	f_Prevotellaceae	0.378	0.048	0.2668
C4	g_Oribacterium	-0.447	0.017	0.1454
PNI	g_Bifidobacterium	0.470	0.012	0.0497
PNI	g_Anaerosporobacter	0.393	0.038	0.2174

Abbreviations: IL-1β, interleukin-1beta; WBC, white blood cell; CRP, C-reactive protein; Alb, albumin; ALT, alanine aminotransferase; AST, aspartate aminotransferase; TC, total cholesterol; TG, triglyceride; HDL, high-density lipoprotein; LDL, low- density lipoprotein; C3, complement-3; C4, complement-4; PNI, prognostic nutritional index; FDR, false discovery rate.
