# Supplementary material for: Correlation between gut microbiome and cognitive impairment in patients undergoing peritoneal dialysis
Source: BMC Nephrol. 2023 Dec 5;24:360. doi: 10.1186/s12882-023-03410-z (PMC10696889; doi:10.1186/s12882-023-03410-z)
Supplement: Supplementary file 7 — Additional file 7: Table S6. The calculation results (unadjusted, and age-adjusted) of the correlation between gut microbiota and cognitive function. [file 12882_2023_3410_MOESM7_ESM.pdf]

**Table S6.** The calculation results (unadjusted, and age-adjusted) of the correlation between gut microbiota and cognitive function.

| | MoCA | | MMSE | | SAS | | SDS | | HAMA | | HAMD | |
|---|---|---|---|---|---|---|---|---|---|---|---|---|
| **Unadjusted** | raw r | raw P | raw r | raw P | raw r | raw P | raw r | raw P | raw r | raw P | raw r | raw P |
| Prevotellaceae | -0.387 | 0.042 | -0.154 | 0.435 | 0.136 | 0.492 | 0.240 | 0.220 | 0.196 | 0.317 | 0.468 | 0.012 |
| Actinomycetaceae | 0.446 | 0.017 | 0.353 | 0.065 | -0.121 | 0.540 | -0.039 | 0.844 | -0.199 | 0.310 | -0.278 | 0.153 |
| Lactobacillaceae | 0.387 | 0.042 | 0.213 | 0.276 | 0.188 | 0.337 | 0.261 | 0.180 | -0.006 | 0.977 | -0.052 | 0.792 |
| Propionibacteriaceae | 0.339 | 0.078 | 0.222 | 0.257 | -0.180 | 0.360 | -0.280 | 0.150 | -0.356 | 0.063 | -0.421 | 0.026 |
| Streptococcaceae | 0.367 | 0.054 | 0.318 | 0.100 | -0.102 | 0.606 | -0.030 | 0.878 | 0.033 | 0.869 | -0.114 | 0.563 |
| Actinomyces | 0.458 | 0.014 | 0.532 | 0.004 | -0.241 | 0.217 | 0.070 | 0.723 | -0.010 | 0.961 | -0.095 | 0.632 |
| Streptococcus | 0.367 | 0.054 | 0.395 | 0.038 | -0.088 | 0.656 | 0.008 | 0.968 | 0.129 | 0.512 | -0.032 | 0.873 |
| Oribacterium | 0.351 | 0.067 | 0.487 | 0.009 | -0.196 | 0.317 | -0.177 | 0.367 | -0.248 | 0.204 | -0.342 | 0.075 |
| Atopobium | 0.471 | 0.011 | 0.416 | 0.028 | 0.008 | 0.968 | -0.021 | 0.917 | -0.055 | 0.780 | -0.164 | 0.406 |
| Lactobacillus | 0.461 | 0.014 | 0.264 | 0.175 | 0.187 | 0.349 | 0.183 | 0.350 | 0.016 | 0.935 | -0.048 | 0.808 |
| **Age-adjusted** | adj r | adj P | adj r | adj P | adj r | adj P | adj r | adj P | adj r | adj P | adj r | adj P |
| Prevotellaceae | -0.267 | 0.178 | -0.247 | 0.214 | -0.260 | 0.896 | 0.110 | 0.586 | -0.063 | 0.754 | 0.019 | 0.925 |
| Actinomycetaceae | 0.134 | 0.504 | 0.105 | 0.602 | -0.259 | 0.192 | -0.153 | 0.445 | -0.201 | 0.314 | -0.242 | 0.224 |
| Lactobacillaceae | 0.180 | 0.368 | 0.122 | 0.544 | 0.286 | 0.149 | 0.300 | 0.128 | 0.031 | 0.876 | 0.251 | 0.206 |
| Propionibacteriaceae | 0.122 | 0.545 | 0.940 | 0.640 | -0.256 | 0.197 | -0.175 | 0.383 | -0.218 | 0.275 | 0.246 | 0.215 |
| Streptococcaceae | 0.400 | 0.039 | 0.309 | 0.117 | -0.232 | 0.244 | -0.243 | 0.222 | 0.260 | 0.191 | -0.406 | 0.036 |
| Actinomyces | 0.127 | 0.526 | 0.113 | 0.574 | -0.255 | 0.199 | -0.143 | 0.475 | -0.177 | 0.376 | -0.215 | 0.282 |
| Streptococcus | 0.339 | 0.084 | 0.386 | 0.047 | -0.196 | 0.326 | -0.165 | 0.411 | -0.030 | 0.880 | -0.170 | 0.397 |
| Oribacterium | 0.133 | 0.508 | 0.129 | 0.521 | -0.230 | 0.249 | -0.153 | 0.446 | -0.140 | 0.487 | -0.187 | 0.351 |
| Atopobium | 0.443 | 0.021 | 0.338 | 0.084 | -0.092 | 0.648 | -0.138 | 0.492 | -0.106 | 0.599 | -0.289 | 0.143 |
| Lactobacillus | 0.187 | 0.349 | 0.125 | 0.543 | 0.287 | 0.146 | 0.294 | 0.136 | 0.030 | 0.883 | 0.247 | 0.215 |

Abbreviations: MoCA, Montreal cognitive assessment scale; MMSE, Mini-mental state examination; SAS, Self-rating anxiety scale; SDS, Self-rating depression scale; HAMA, Hamilton anxiety scale; HAMD, Hamilton depression scale.
