# Supplementary material for: Correlation between gut microbiome and cognitive impairment in patients undergoing peritoneal dialysis
Source: BMC Nephrol. 2023 Dec 5;24:360. doi: 10.1186/s12882-023-03410-z (PMC10696889; doi:10.1186/s12882-023-03410-z)
Supplement: Supplementary file 8 — Additional file 8: Table S7. The KEGG analysis between PD and ESRD. [file 12882_2023_3410_MOESM8_ESM.pdf]

Table S7. The KEGG analysis between PD and ESRD.

Level	KEGG Ortholog	Mean (ESRD)	SD (ESRD)	Mean (PD)	SD (PD)	P value (ESRD vs PD)
Ko level 1	Cellular Processes	3.4364	0.9364	2.9950	0.8415	0.049
	Environmental Information Processing	2.5151	0.5309	2.6882	0.9445	0.994
	Genetic Information Processing	13.8395	0.9053	13.3187	0.9898	0.032
	Human Diseases	0.3583	0.1727	0.4212	0.2314	0.267
	Metabolism	79.4032	1.4971	80.1402	1.5675	0.127
	Organismal Systems	0.4475	0.0481	0.4368	0.0579	0.487
Ko level 2	Amino acid metabolism	12.9780	0.7296	12.4127	1.1665	0.002
	Energy metabolism	5.4425	0.3196	5.4776	0.2805	0.768
	Environmental adaptation	0.2061	0.0380	0.1979	0.0341	0.439
	Excretory system	0.0000	0.0000	0.0000	0.0000	0.326
	Folding, sorting and degradation	3.2293	0.2037	3.0675	0.1764	0.005
	Glycan biosynthesis and metabolism	5.1124	1.2120	5.6568	1.5156	0.216
	Immune diseases	0.0000	0.0000	0.0000	0.0000	0.350
	Immune system	0.0874	0.0142	0.0903	0.0255	0.539
	Infectious diseases: Bacterial	0.3423	0.1696	0.4023	0.2220	0.318
	Infectious diseases: Parasitic	0.0147	0.0094	0.0172	0.0134	0.604
	Lipid metabolism	5.7580	0.7309	5.9019	1.1606	0.792
	Biosynthesis of other secondary metabolites	2.2279	0.2191	2.2750	0.2673	0.140
	Membrane transport	2.1201	0.4690	2.2997	0.8931	0.905
	Metabolism of cofactors and vitamins	13.1137	1.1401	13.2423	1.5579	0.879
	Metabolism of other amino acids	6.6024	0.4928	6.7020	0.5879	0.393
	Metabolism of terpenoids and polyketides	9.5101	1.0596	9.0902	0.9463	0.539

	Neurodegenerative diseases	0.0009	0.0022	0.0009	0.0009	0.093
	Nucleotide metabolism	2.0740	0.0942	2.0488	0.1403	0.411
	Replication and repair	6.1899	0.3600	6.0709	0.5526	0.274
	Signal transduction	0.3950	0.1084	0.3885	0.1213	0.649
	Signaling molecules and interaction	0.0000	0.0000	0.0000	0.0000	0.087
	Transcription	1.1003	0.2086	0.9951	0.2031	0.092
	Carbohydrate metabolism	15.4573	1.2164	15.7518	1.2894	0.350
	Translation	3.3200	0.2937	3.1853	0.3296	0.080
	Transport and catabolism	0.2120	0.0488	0.2173	0.0602	0.429
	Xenobiotics biodegradation and metabolism	1.1270	0.5241	1.5812	0.8629	0.017
	Cardiovascular diseases	0.0005	0.0008	0.0008	0.0021	0.929
	Cell growth and death	1.5545	0.1439	1.4865	0.1525	0.095
	Cell motility	1.6699	0.9798	1.2912	0.8578	0.089
	Cellular community	0.0000	0.0000	0.0000	0.0000	0.564
	Digestive system	0.0460	0.0318	0.0481	0.0321	0.892
	Endocrine system	0.1080	0.0203	0.1005	0.0224	0.228
Ko level 3	ABC transporters	0.7717	0.1775	0.7667	0.1635	0.792
	Ascorbate and aldarate metabolism	0.2616	0.1066	0.3476	0.1768	0.015
	Photosynthesis	0.1168	0.2343	0.1288	0.2296	0.845
	Photosynthesis - antenna proteins	0.0000	0.0000	0.0000	0.0001	0.664
	Plant hormone signal transduction	0.0003	0.0006	0.0003	0.0006	0.620
	Plant-pathogen interaction	0.2061	0.0380	0.1979	0.0341	0.439
	Polyketide sugar unit biosynthesis	0.3156	0.2560	0.2822	0.2713	0.765
	Porphyrin and chlorophyll metabolism	0.7786	0.0937	0.7270	0.2082	0.350
	Primary bile acid biosynthesis	0.1355	0.0604	0.1504	0.0888	0.571

Propanoate metabolism	0.6803	0.0929	0.6885	0.0856	0.792
Proteasome	0.0013	0.0056	0.0001	0.0003	0.103
Protein digestion and absorption	0.0460	0.0318	0.0481	0.0321	0.892
Atrazine degradation	0.0298	0.0468	0.0314	0.0359	0.604
Protein export	1.4666	0.1034	1.3907	0.0919	0.002
Protein processing in endoplasmic reticulum	0.0521	0.0126	0.0495	0.0111	0.281
Purine metabolism	0.8870	0.0379	0.8815	0.0508	0.661
Pyrimidine metabolism	1.1870	0.0645	1.1673	0.0950	0.448
Pyruvate metabolism	1.1691	0.1060	1.1956	0.1091	0.487
RNA degradation	0.6250	0.0473	0.6259	0.0469	0.690
RNA polymerase	1.1003	0.2085	0.9950	0.2031	0.092
RNA transport	0.0540	0.0080	0.0510	0.0100	0.234
Renin-angiotensin system	0.0000	0.0000	0.0000	0.0000	0.154
Retinol metabolism	0.0306	0.0338	0.0314	0.0354	0.907
Bacterial chemotaxis	1.0393	0.5865	0.7890	0.4780	0.073
Riboflavin metabolism	0.9514	0.1400	0.9135	0.2177	0.571
Ribosome	1.5585	0.1608	1.5005	0.1731	0.120
Ribosome biogenesis in eukaryotes	0.0573	0.0163	0.0537	0.0045	0.518
Secondary bile acid biosynthesis	1.2195	0.5436	1.3532	0.7991	0.571
Selenocompound metabolism	1.0663	0.0700	1.1171	0.0881	0.025
Shigellosis	0.0317	0.0543	0.0585	0.0905	0.061
Sphingolipid metabolism	0.5125	0.1798	0.5615	0.2015	0.719
Spliceosome	0.0000	0.0000	0.0000	0.0000	0.763
Staphylococcus aureus infection	0.0127	0.0193	0.0226	0.0815	0.731
Starch and sucrose metabolism	1.2020	0.1760	1.1968	0.1260	0.792

Bacterial invasion of epithelial cells	0.0324	0.0550	0.0596	0.0916	0.063
Steroid biosynthesis	0.0045	0.0153	0.0108	0.0310	0.416
Steroid hormone biosynthesis	0.0437	0.0327	0.0537	0.0323	0.216
Streptomycin biosynthesis	1.7100	0.1808	1.7527	0.2591	0.131
Styrene degradation	0.0093	0.0498	0.0157	0.0478	0.096
Sulfur metabolism	0.8479	0.0953	0.8915	0.1278	0.102
Sulfur relay system	1.0843	0.1453	1.0012	0.1684	0.086
Synthesis and degradation of ketone bodies	0.2939	0.1710	0.2749	0.1955	0.593
Systemic lupus erythematosus	0.0000	0.0000	0.0000	0.0000	0.350
Taurine and hypotaurine metabolism	0.6026	0.1014	0.6570	0.1058	0.080
Terpenoid backbone biosynthesis	1.3160	0.1150	1.2548	0.1339	0.056
Bacterial secretion system	0.8413	0.1082	0.8219	0.1082	0.318
Thiamine metabolism	1.6883	0.1976	1.6023	0.1604	0.112
Tropane, piperidine and pyridine alkaloid biosynthesis	0.4417	0.1248	0.4474	0.1503	0.566
Tryptophan metabolism	0.1475	0.0584	0.1292	0.0784	1.000
Two-component system	0.3947	0.1080	0.3881	0.1213	0.649
Tyrosine metabolism	0.2744	0.0424	0.2766	0.0280	0.204
Ubiquinone and other terpenoid-quinone biosynthesis	0.3585	0.1528	0.4532	0.2112	0.086
Valine, leucine and isoleucine biosynthesis	2.3462	0.1808	2.2226	0.2246	0.068
Valine, leucine and isoleucine degradation	0.3529	0.0733	0.3892	0.0801	0.216
Vasopressin-regulated water reabsorption	0.0000	0.0000	0.0000	0.0000	0.326
Vibrio cholerae infection	0.0000	0.0000	0.0001	0.0004	0.237
Basal transcription factors	0.0000	0.0002	0.0001	0.0003	0.463
Vibrio cholerae pathogenic cycle	0.1026	0.0870	0.1051	0.0888	0.902
Vitamin B6 metabolism	1.2216	0.1656	1.1508	0.2335	0.116

Xylene degradation	0.0054	0.0291	0.0223	0.0663	0.272
Zeatin biosynthesis	0.6631	0.1223	0.6652	0.1408	0.756
beta-Alanine metabolism	0.1305	0.2189	0.1882	0.2478	0.332
mRNA surveillance pathway	0.0000	0.0000	0.0000	0.0001	0.463
Base excision repair	0.9303	0.0740	0.9307	0.0988	0.638
Benzoate degradation	0.1757	0.0351	0.1677	0.0451	0.167
Biosynthesis of ansamycins	4.8824	1.2123	4.4718	0.9242	0.518
Biosynthesis of siderophore group nonribosomal peptides	0.0853	0.1116	0.1138	0.0894	0.063
African trypanosomiasis	0.0060	0.0096	0.0087	0.0119	0.376
Biosynthesis of unsaturated fatty acids	0.3562	0.0557	0.3468	0.0839	0.768
Biosynthesis of vancomycin group antibiotics	2.2193	0.2144	2.2249	0.4004	0.743
Biotin metabolism	1.4486	0.3077	1.5684	0.3728	0.116
Butanoate metabolism	0.7163	0.1090	0.7231	0.1204	0.672
C5-Branched dibasic acid metabolism	1.9049	0.2126	1.8227	0.3027	0.498
Calcium signaling pathway	0.0000	0.0000	0.0001	0.0004	0.926
Caprolactam degradation	0.0254	0.0374	0.0402	0.0619	0.402
Carbon fixation in photosynthetic organisms	1.6633	0.1539	1.6517	0.0963	0.756
Carbon fixation pathways in prokaryotes	1.1475	0.1339	1.1390	0.1849	0.917
Carotenoid biosynthesis	0.0042	0.0074	0.0070	0.0164	0.774
Alanine, aspartate and glutamate metabolism	1.8194	0.2015	1.7238	0.1654	0.099
Cell cycle - Caulobacter	1.5252	0.1313	1.4544	0.1418	0.059
Chagas disease (American trypanosomiasis)	0.0005	0.0025	0.0003	0.0015	0.578
Chloroalkane and chloroalkene degradation	0.0000	0.0000	0.0511	0.1506	0.076
Chlorocyclohexane and chlorobenzene degradation	0.0646	0.0328	0.0814	0.0513	0.183
Citrate cycle (TCA cycle)	1.0326	0.1822	1.0533	0.2091	0.478

Cysteine and methionine metabolism	1.3872	0.0972	1.3431	0.1713	0.059
D-Alanine metabolism	1.7381	0.1405	1.7417	0.1934	0.981
D-Arginine and D-ornithine metabolism	0.2946	0.2529	0.2569	0.3127	0.274
D-Glutamine and D-glutamate metabolism	2.1054	0.1957	2.0307	0.2620	0.274
DNA replication	1.2184	0.0778	1.1991	0.1255	0.296
Amino sugar and nucleotide sugar metabolism	1.1885	0.1087	1.2541	0.2425	0.604
Dioxin degradation	0.0545	0.1180	0.0681	0.1436	0.761
Drug metabolism - other enzymes	0.1443	0.4329	0.4648	0.6425	0.036
ECM-receptor interaction	0.0000	0.0000	0.0000	0.0000	0.087
Endocytosis	0.0005	0.0007	0.0004	0.0008	0.539
Epithelial cell signaling in Helicobacter pylori infection	0.1629	0.0179	0.1565	0.0224	0.127
Fatty acid biosynthesis	1.6357	0.1611	1.6100	0.2257	0.487
Fatty acid degradation	0.3411	0.0654	0.3467	0.0558	0.528
Flagellar assembly	0.6307	0.4110	0.5022	0.3946	0.112
Flavonoid biosynthesis	0.0132	0.0156	0.0147	0.0197	0.879
Fluorobenzoate degradation	0.0006	0.0031	0.0000	0.0000	0.343
Aminoacyl-tRNA biosynthesis	1.6501	0.1356	1.5800	0.1637	0.083
Focal adhesion	0.0000	0.0000	0.0000	0.0000	0.564
Folate biosynthesis	1.0969	0.2635	1.1742	0.2729	0.267
Fructose and mannose metabolism	1.1883	0.2827	1.2990	0.4420	0.281
Galactose metabolism	1.2873	0.2289	1.3108	0.2860	0.879
Geraniol degradation	0.0241	0.0547	0.0705	0.1272	0.295
Glutathione metabolism	0.4810	0.1303	0.5215	0.2012	0.508
Glycerolipid metabolism	0.5475	0.1280	0.5189	0.1016	0.508
Glycerophospholipid metabolism	0.5880	0.0637	0.6075	0.0872	0.518

Glycine, serine and threonine metabolism	1.1436	0.0661	1.1017	0.1113	0.043
Glycolysis / Gluconeogenesis	1.1325	0.1021	1.1926	0.2171	0.222
Aminobenzoate degradation	0.1111	0.0428	0.1323	0.0547	0.204
Glycosaminoglycan degradation	0.5561	0.3847	0.7884	0.5753	0.144
Glycosphingolipid biosynthesis - lacto and neolacto series	0.0008	0.0017	0.0009	0.0015	0.844
Glyoxylate and dicarboxylate metabolism	0.7615	0.0672	0.7701	0.1486	0.672
Histidine metabolism	1.2788	0.1931	1.1522	0.2843	0.002
Homologous recombination	1.5316	0.1089	1.4856	0.1220	0.260
Hypertrophic cardiomyopathy (HCM)	0.0005	0.0008	0.0008	0.0021	0.929
Inositol phosphate metabolism	0.2137	0.0643	0.2058	0.0385	0.805
Insulin signaling pathway	0.1080	0.0203	0.1005	0.0224	0.228
Isoflavonoid biosynthesis	0.0000	0.0000	0.0001	0.0004	0.154
Linoleic acid metabolism	0.0799	0.1572	0.0677	0.1345	0.975
Amoebiasis	0.0082	0.0052	0.0082	0.0062	0.719
Lipoic acid metabolism	0.7739	0.3571	0.9578	0.4520	0.144
Lipopolysaccharide biosynthesis	0.9206	0.4433	1.0526	0.4559	0.303
Lysine biosynthesis	1.6654	0.1242	1.5958	0.2440	0.024
Lysine degradation	0.1684	0.0242	0.1767	0.0433	0.487
Meiosis - yeast	0.0000	0.0000	0.0000	0.0001	0.148
Methane metabolism	0.5100	0.0587	0.5067	0.0516	0.743
Mismatch repair	1.6938	0.0950	1.6712	0.1596	0.518
N-Glycan biosynthesis	0.0474	0.0211	0.0497	0.0256	0.661
NOD-like receptor signaling pathway	0.0874	0.0142	0.0903	0.0255	0.539
Naphthalene degradation	0.1579	0.1245	0.1478	0.1380	0.902
Apoptosis	0.0293	0.0231	0.0321	0.0256	0.731

Nicotinate and nicotinamide metabolism	1.1043	0.0967	1.0855	0.1204	0.539
Nitrogen metabolism	0.6836	0.1474	0.6833	0.1078	0.458
Nitrotoluene degradation	0.3486	0.1525	0.3583	0.1853	0.571
Non-homologous end-joining	0.0187	0.0344	0.0120	0.0127	0.943
Nucleotide excision repair	0.7970	0.0583	0.7723	0.0905	0.318
One carbon pool by folate	1.7868	0.1316	1.7566	0.1364	0.311
Other glycan degradation	1.6792	0.6798	1.9163	0.9969	0.684
Other types of O-glycan biosynthesis	0.0000	0.0001	0.0000	0.0001	0.915
Oxidative phosphorylation	0.4733	0.0339	0.4765	0.0546	0.768
Pantothenate and CoA biosynthesis	1.8743	0.1245	1.8216	0.1380	0.105
Arginine and proline metabolism	0.7801	0.0488	0.7467	0.0824	0.109
Parkinson's disease	0.0009	0.0022	0.0009	0.0009	0.093
Penicillin and cephalosporin biosynthesis	0.0629	0.0368	0.0602	0.0411	0.792
Pentose and glucuronate interconversions	0.8839	0.1822	0.8904	0.2178	0.842
Pentose phosphate pathway	1.8348	0.2679	1.8015	0.2140	0.756
Peptidoglycan biosynthesis	1.9083	0.1787	1.8488	0.2561	0.177
Peroxisome	0.2115	0.0491	0.2168	0.0603	0.402
Phenylalanine metabolism	0.3219	0.0747	0.3275	0.0597	0.376
Phenylalanine, tyrosine and tryptophan biosynthesis	1.2921	0.1029	1.2276	0.1582	0.086
Phosphonate and phosphinate metabolism	0.1839	0.0573	0.1890	0.0783	0.549
Phosphotransferase system (PTS)	0.5071	0.3284	0.7111	0.8267	0.649

Abbreviations: ESRD, end stage renal disease; PD, peritoneal dialysis; Ko, KEGG Ortholog.
