# Supplementary material for: Correlation between gut microbiome and cognitive impairment in patients undergoing peritoneal dialysis
Source: BMC Nephrol. 2023 Dec 5;24:360. doi: 10.1186/s12882-023-03410-z (PMC10696889; doi:10.1186/s12882-023-03410-z)
Supplement: Supplementary file 9 — Additional file 9: Table S8. The KEGG analysis between PCI and PNCI. [file 12882_2023_3410_MOESM9_ESM.pdf]

Table S8. The KEGG analysis between PCI and PNCI.

Level	KEGG Ortholog	Mean (PCI)	SD (PCI)	Mean (PNCI)	SD (PNCI)	P value (PCI vs PNCI)
Ko level 1	Cellular Processes	3.0725	0.8675	2.8313	0.8077	0.491
	Environmental Information Processing	2.6983	0.7484	2.6668	1.3229	0.403
	Genetic Information Processing	13.1396	0.9285	13.6969	1.0628	0.238
	Human Diseases	0.4219	0.2507	0.4196	0.1985	1.000
	Metabolism	80.2228	1.5027	79.9657	1.7780	0.844
	Organismal Systems	0.4449	0.0455	0.4197	0.0786	0.658
Ko level 2	Amino acid metabolism	12.5423	0.7940	12.1390	1.7464	0.961
	Energy metabolism	5.4669	0.2485	5.5001	0.3547	0.731
	Environmental adaptation	0.1994	0.0340	0.1947	0.0362	0.768
	Excretory system	0.0000	0.0000	0.0000	0.0000	0.541
	Folding, sorting and degradation	3.0584	0.1764	3.0865	0.1856	0.844
	Glycan biosynthesis and metabolism	5.6959	1.3131	5.5741	1.9652	0.491
	Immune diseases	0.0000	0.0000	0.0000	0.0000	0.131
	Immune system	0.0922	0.0146	0.0861	0.0410	0.279
	Infectious diseases: Bacterial	0.4021	0.2389	0.4026	0.1946	0.961
	Infectious diseases: Parasitic	0.0182	0.0138	0.0150	0.0130	0.431
	Lipid metabolism	5.7303	0.8094	6.2643	1.6894	0.623
	Biosynthesis of other secondary metabolites	2.3183	0.2671	2.1836	0.2582	0.094
	Membrane transport	2.2960	0.7026	2.3074	1.2573	0.376
	Metabolism of cofactors and vitamins	13.1957	1.1697	13.3406	2.2575	0.768
	Metabolism of other amino acids	6.5982	0.5499	6.9212	0.6375	0.184

	Metabolism of terpenoids and polyketides	9.2379	0.8541	8.7782	1.1044	0.350
	Neurodegenerative diseases	0.0010	0.0010	0.0007	0.0006	0.675
	Nucleotide metabolism	2.0310	0.1333	2.0863	0.1553	0.376
	Replication and repair	5.9493	0.4764	6.3275	0.6408	0.154
	Signal transduction	0.4023	0.1208	0.3594	0.1243	0.279
	Transcription	0.9930	0.2069	0.9995	0.2071	0.883
	Carbohydrate metabolism	15.8392	1.0243	15.5671	1.7872	0.140
	Translation	3.1389	0.3227	3.2833	0.3412	0.279
	Transport and catabolism	0.2131	0.0523	0.2260	0.0770	0.694
	Xenobiotics biodegradation and metabolism	1.5670	0.6650	1.6112	1.2315	0.555
	Cardiovascular diseases	0.0005	0.0009	0.0014	0.0035	0.881
	Cell growth and death	1.4648	0.1550	1.5325	0.1447	0.258
	Cell motility	1.3946	0.8444	1.0728	0.8946	0.350
	Cellular community	0.0000	0.0000	0.0000	0.0000	0.169
	Digestive system	0.0477	0.0252	0.0491	0.0453	0.883
	Endocrine system	0.1055	0.0207	0.0899	0.0233	0.192
Ko level 3	ABC transporters	0.7706	0.1477	0.7585	0.2025	1.000
	Ascorbate and aldarate metabolism	0.3343	0.1190	0.3757	0.2689	0.431
	Photosynthesis	0.1036	0.2090	0.1821	0.2738	0.400
	Photosynthesis - antenna proteins	0.0000	0.0001	0.0001	0.0002	0.518
	Plant hormone signal transduction	0.0003	0.0006	0.0004	0.0006	0.362
	Plant-pathogen interaction	0.1994	0.0340	0.1947	0.0362	0.768
	Polyketide sugar unit biosynthesis	0.2588	0.2825	0.3315	0.2544	0.795
	Porphyrin and chlorophyll metabolism	0.7413	0.1783	0.6969	0.2708	0.588
	Primary bile acid biosynthesis	0.1361	0.0746	0.1804	0.1123	0.523

Propanoate metabolism	0.6959	0.0976	0.6729	0.0541	0.731
Proteasome	0.0001	0.0003	0.0002	0.0003	0.027
Protein digestion and absorption	0.0477	0.0252	0.0491	0.0453	0.883
Atrazine degradation	0.0283	0.0242	0.0382	0.0545	0.539
Protein export	1.3804	0.0973	1.4126	0.0799	0.127
Protein processing in endoplasmic reticulum	0.0505	0.0099	0.0475	0.0138	0.806
Purine metabolism	0.8733	0.0510	0.8987	0.0485	0.279
Pyrimidine metabolism	1.1577	0.0879	1.1876	0.1114	0.403
Pyruvate metabolism	1.1996	0.1109	1.1872	0.1114	0.961
RNA degradation	0.6201	0.0484	0.6381	0.0439	0.219
RNA polymerase	0.9929	0.2068	0.9994	0.2071	0.883
RNA transport	0.0508	0.0102	0.0513	0.0100	1.000
Renin-angiotensin system	0.0000	0.0000	0.0000	0.0000	0.582
Retinol metabolism	0.0387	0.0391	0.0158	0.0196	0.147
Bacterial chemotaxis	0.8395	0.4525	0.6822	0.5399	0.302
Riboflavin metabolism	0.9248	0.1912	0.8895	0.2770	0.961
Ribosome	1.4701	0.1612	1.5646	0.1893	0.154
Ribosome biogenesis in eukaryotes	0.0536	0.0047	0.0541	0.0043	0.768
Secondary bile acid biosynthesis	1.2250	0.6710	1.6236	1.0104	0.523
Selenocompound metabolism	1.1167	0.0829	1.1178	0.1036	0.844
Shigellosis	0.0701	0.1028	0.0340	0.0532	0.376
Sphingolipid metabolism	0.5716	0.1934	0.5402	0.2281	0.623
Spliceosome	0.0000	0.0000	0.0000	0.0000	0.808
Staphylococcus aureus infection	0.0082	0.0103	0.0529	0.1437	0.883
Starch and sucrose metabolism	1.2131	0.0870	1.1624	0.1861	0.258

Bacterial invasion of epithelial cells	0.0714	0.1039	0.0347	0.0544	0.350
Steroid biosynthesis	0.0122	0.0361	0.0080	0.0174	0.125
Steroid hormone biosynthesis	0.0564	0.0315	0.0478	0.0350	0.461
Streptomycin biosynthesis	1.7729	0.2911	1.7099	0.1812	0.127
Styrene degradation	0.0079	0.0235	0.0323	0.0775	0.659
Sulfur metabolism	0.9080	0.1221	0.8568	0.1398	0.350
Sulfur relay system	1.0073	0.1799	0.9882	0.1503	0.806
Synthesis and degradation of ketone bodies	0.2458	0.1330	0.3364	0.2879	0.491
Systemic lupus erythematosus	0.0000	0.0000	0.0000	0.0000	0.131
Taurine and hypotaurine metabolism	0.6708	0.1134	0.6279	0.0863	0.279
Terpenoid backbone biosynthesis	1.2367	0.1230	1.2931	0.1551	0.403
Bacterial secretion system	0.8346	0.1162	0.7949	0.0889	0.623
Thiamine metabolism	1.6008	0.1519	1.6053	0.1870	0.731
Tropane, piperidine and pyridine alkaloid biosynthesis	0.4638	0.1465	0.4129	0.1612	0.461
Tryptophan metabolism	0.1141	0.0823	0.1610	0.0620	0.026
Two-component system	0.4020	0.1207	0.3587	0.1241	0.258
Tyrosine metabolism	0.2849	0.0225	0.2591	0.0316	0.044
Ubiquinone and other terpenoid-quinone biosynthesis	0.4547	0.2278	0.4499	0.1840	0.961
Valine, leucine and isoleucine biosynthesis	2.2024	0.1679	2.2653	0.3221	0.184
Valine, leucine and isoleucine degradation	0.3828	0.0907	0.4025	0.0535	0.461
Vasopressin-regulated water reabsorption	0.0000	0.0000	0.0000	0.0000	0.541
Vibrio cholerae infection	0.0001	0.0004	0.0000	0.0000	0.451
Basal transcription factors	0.0001	0.0003	0.0001	0.0002	0.660
Vibrio cholerae pathogenic cycle	0.0943	0.0930	0.1278	0.0791	0.247
Vitamin B6 metabolism	1.1947	0.1697	1.0580	0.3240	0.350

Xylene degradation	0.0217	0.0659	0.0236	0.0709	1.000
Zeatin biosynthesis	0.6466	0.1116	0.7045	0.1906	0.523
beta-Alanine metabolism	0.1476	0.2276	0.2739	0.2801	0.199
mRNA surveillance pathway	0.0000	0.0001	0.0000	0.0000	0.660
Base excision repair	0.9131	0.0607	0.9677	0.1495	0.302
Benzoate degradation	0.1639	0.0379	0.1755	0.0595	0.844
Biosynthesis of ansamycins	4.5794	0.7805	4.2446	1.1943	0.623
Biosynthesis of siderophore group nonribosomal peptides	0.1198	0.0968	0.1010	0.0748	0.806
African trypanosomiasis	0.0098	0.0137	0.0063	0.0071	0.694
Biosynthesis of unsaturated fatty acids	0.3498	0.0866	0.3406	0.0826	0.844
Biosynthesis of vancomycin group antibiotics	2.2967	0.4095	2.0734	0.3546	0.258
Biotin metabolism	1.5737	0.3096	1.5572	0.5031	1.000
Butanoate metabolism	0.7376	0.1347	0.6923	0.0809	0.555
C5-Branched dibasic acid metabolism	1.8145	0.1683	1.8401	0.4950	0.154
Calcium signaling pathway	0.0001	0.0003	0.0002	0.0007	0.659
Caprolactam degradation	0.0473	0.0702	0.0250	0.0383	0.491
Carbon fixation in photosynthetic organisms	1.6546	0.0833	1.6456	0.1250	0.555
Carbon fixation pathways in prokaryotes	1.1369	0.1284	1.1434	0.2798	0.376
Carotenoid biosynthesis	0.0082	0.0192	0.0047	0.0081	0.555
Alanine, aspartate and glutamate metabolism	1.7509	0.1373	1.6667	0.2108	0.431
Cell cycle - Caulobacter	1.4352	0.1471	1.4952	0.1281	0.258
Chagas disease (American trypanosomiasis)	0.0004	0.0018	0.0000	0.0000	0.349
Chloroalkane and chloroalkene degradation	0.0234	0.1019	0.1098	0.2178	0.169
Chlorocyclohexane and chlorobenzene degradation	0.0818	0.0459	0.0807	0.0644	0.768
Citrate cycle (TCA cycle)	1.0596	0.1967	1.0399	0.2454	0.922

Cysteine and methionine metabolism	1.3582	0.1774	1.3113	0.1629	0.844
D-Alanine metabolism	1.7181	0.1658	1.7915	0.2456	0.376
D-Arginine and D-ornithine metabolism	0.2307	0.3320	0.3121	0.2773	0.201
D-Glutamine and D-glutamate metabolism	2.0125	0.2734	2.0691	0.2471	0.588
DNA replication	1.1714	0.1134	1.2574	0.1361	0.069
Amino sugar and nucleotide sugar metabolism	1.2699	0.2357	1.2205	0.2676	0.094
Dioxin degradation	0.0486	0.1051	0.1092	0.2049	0.438
Drug metabolism - other enzymes	0.4870	0.6645	0.4180	0.6292	0.774
Endocytosis	0.0006	0.0009	0.0002	0.0002	0.605
Epithelial cell signaling in Helicobacter pylori infection	0.1581	0.0163	0.1532	0.0328	0.844
Fatty acid biosynthesis	1.5848	0.1982	1.6634	0.2807	0.523
Fatty acid degradation	0.3570	0.0586	0.3248	0.0449	0.168
Flagellar assembly	0.5550	0.4064	0.3906	0.3652	0.258
Flavonoid biosynthesis	0.0182	0.0226	0.0071	0.0075	0.140
Aminoacyl-tRNA biosynthesis	1.5643	0.1657	1.6133	0.1638	0.588
Focal adhesion	0.0000	0.0000	0.0000	0.0000	0.169
Folate biosynthesis	1.1591	0.2145	1.2060	0.3824	0.806
Fructose and mannose metabolism	1.2838	0.3452	1.3309	0.6241	0.431
Galactose metabolism	1.3332	0.2560	1.2634	0.3533	0.922
Geraniol degradation	0.0918	0.1423	0.0255	0.0758	0.148
Glutathione metabolism	0.5246	0.2233	0.5151	0.1561	0.961
Glycerolipid metabolism	0.5270	0.0911	0.5017	0.1254	0.431
Glycerophospholipid metabolism	0.6157	0.0923	0.5902	0.0777	0.588
Glycine, serine and threonine metabolism	1.1192	0.0736	1.0647	0.1655	0.623
Glycolysis / Gluconeogenesis	1.1872	0.1372	1.2041	0.3413	0.140

Aminobenzoate degradation	0.1416	0.0577	0.1128	0.0445	0.140
Glycosaminoglycan degradation	0.8093	0.5896	0.7443	0.5758	0.731
Glycosphingolipid biosynthesis - lacto and neolacto series	0.0010	0.0017	0.0006	0.0011	0.701
Glyoxylate and dicarboxylate metabolism	0.7938	0.1414	0.7199	0.1592	0.403
Histidine metabolism	1.1988	0.1968	1.0538	0.4118	0.555
Homologous recombination	1.4644	0.1130	1.5305	0.1348	0.302
Hypertrophic cardiomyopathy (HCM)	0.0005	0.0009	0.0014	0.0035	0.881
Inositol phosphate metabolism	0.2103	0.0409	0.1964	0.0333	0.238
Insulin signaling pathway	0.1055	0.0207	0.0899	0.0233	0.192
Isoflavonoid biosynthesis	0.0001	0.0005	0.0000	0.0000	0.660
Linoleic acid metabolism	0.0490	0.1201	0.1072	0.1613	0.155
Amoebiasis	0.0080	0.0059	0.0087	0.0072	0.883
Lipoic acid metabolism	0.8866	0.4559	1.1082	0.4293	0.325
Lipopolysaccharide biosynthesis	1.0641	0.4023	1.0284	0.5801	0.961
Lysine biosynthesis	1.6120	0.2678	1.5614	0.1940	0.588
Lysine degradation	0.1874	0.0413	0.1541	0.0407	0.168
Meiosis - yeast	0.0000	0.0000	0.0000	0.0001	0.396
Methane metabolism	0.5010	0.0523	0.5190	0.0510	0.555
Mismatch repair	1.6342	0.1386	1.7494	0.1806	0.140
N-Glycan biosynthesis	0.0453	0.0241	0.0590	0.0276	0.154
NOD-like receptor signaling pathway	0.0922	0.0146	0.0861	0.0410	0.279
Naphthalene degradation	0.1302	0.1466	0.1851	0.1168	0.473
Apoptosis	0.0296	0.0186	0.0373	0.0373	0.806
Nicotinate and nicotinamide metabolism	1.0877	0.1065	1.0807	0.1530	0.623
Nitrogen metabolism	0.6900	0.1003	0.6692	0.1276	0.961

Nitrotoluene degradation	0.3854	0.1735	0.3009	0.2065	0.279
Non-homologous end-joining	0.0098	0.0096	0.0168	0.0173	0.491
Nucleotide excision repair	0.7564	0.0852	0.8058	0.0972	0.325
One carbon pool by folate	1.7478	0.1251	1.7752	0.1644	0.623
Other glycan degradation	1.9416	0.8778	1.8629	1.2711	0.523
Other types of O-glycan biosynthesis	0.0000	0.0001	0.0000	0.0000	0.957
Oxidative phosphorylation	0.4730	0.0491	0.4839	0.0675	0.806
Pantothenate and CoA biosynthesis	1.7855	0.1270	1.8979	0.1357	0.049
Arginine and proline metabolism	0.7607	0.0438	0.7169	0.1309	0.768
Parkinson's disease	0.0010	0.0010	0.0007	0.0006	0.675
Penicillin and cephalosporin biosynthesis	0.0633	0.0464	0.0536	0.0280	0.961
Pentose and glucuronate interconversions	0.8972	0.1552	0.8758	0.3250	0.154
Pentose phosphate pathway	1.8091	0.1760	1.7856	0.2905	0.258
Peptidoglycan biosynthesis	1.8346	0.2516	1.8789	0.2782	0.806
Peroxisome	0.2126	0.0524	0.2259	0.0770	0.694
Phenylalanine metabolism	0.3383	0.0536	0.3047	0.0686	0.302
Phenylalanine, tyrosine and tryptophan biosynthesis	1.2324	0.1180	1.2175	0.2301	0.588
Phosphonate and phosphinate metabolism	0.1772	0.0618	0.2138	0.1051	0.238
Phosphotransferase system (PTS)	0.6908	0.6227	0.7540	1.1962	0.258

Abbreviations: PNCI, peritoneal dialysis patient with normal cognition; PCI, peritoneal dialysis patient with cognitive impairment; Ko, KEGG Ortholog.
