# Supplementary material for: Correlation between gut microbiome and cognitive impairment in patients undergoing peritoneal dialysis
Source: BMC Nephrol. 2023 Dec 5;24:360. doi: 10.1186/s12882-023-03410-z (PMC10696889; doi:10.1186/s12882-023-03410-z)
Supplement: Supplementary file 10 — Additional file 10: Table S9. The MetaCys analysis results. [file 12882_2023_3410_MOESM10_ESM.pdf]

Table S9. The MetaCys analysis results.

MetaCyc pathway	Mean (ESRD)	SD (ESRD)	Mean (PD)	SD (PD)	P value (ESRD vs PD)	Mean (PCI)	SD (PCI)	Mean (PNCI)	SD (PNCI)	P value (PCI vs PNCI)
Alcohol Degradation	0.0588	0.0817	0.0725	0.0768	0.222	0.0821	0.0821	0.0523	0.0637	0.376
Aromatic Compound Degradation	0.0087	0.0231	0.0072	0.0235	0.214	0.0080	0.0284	0.0054	0.0056	0.129
C1 Compound Utilization and Assimilation	1.2339	0.2585	1.2662	0.1895	0.216	1.2544	0.1860	1.2909	0.2058	0.555
Carbohydrate Biosynthesis	4.2293	0.3667	4.1135	0.4543	0.411	4.1456	0.4730	4.0457	0.4308	0.523
Carbohydrate Degradation	3.0858	0.5319	2.9228	0.5444	0.210	2.9044	0.3136	2.9618	0.8812	0.844
Carboxylate Degradation	2.0414	0.3487	2.1579	0.6796	0.593	2.1464	0.3195	2.1822	1.1525	0.127
Cell Structure Biosynthesis	3.1325	0.3320	3.0244	0.4459	0.204	2.8963	0.2516	3.2947	0.6382	0.044
Cofactor,Prosthetic Group,Electron Carrier,and Vitamin Biosynthesis	9.5809	1.0870	9.9253	2.2165	0.095	9.9341	1.9205	9.9068	2.8778	1.000
Degradation/Utilization/Assimilation - Other	0.0077	0.0091	0.0051	0.0124	0.008	0.0060	0.0145	0.0032	0.0064	0.882
Electron Transfer	0.0035	0.0140	0.0014	0.0023	0.981	0.0015	0.0026	0.0011	0.0016	0.786
Fatty Acid and Lipid Biosynthesis	5.8740	1.1672	5.9164	0.7918	0.593	6.0602	0.7054	5.6126	0.9183	0.049
Aldehyde Degradation	0.0209	0.0318	0.0364	0.0481	0.086	0.0429	0.0532	0.0227	0.0334	0.302
Fatty Acid and Lipid Degradation	0.0426	0.0576	0.0731	0.0871	0.095	0.0852	0.0962	0.0476	0.0612	0.302
Fermentation	3.1782	0.5007	3.2908	0.5823	0.696	3.2859	0.5466	3.3012	0.6868	0.961
Glycan Biosynthesis	0.5185	0.1622	0.4201	0.1649	0.065	0.4376	0.1537	0.3832	0.1907	0.731
Glycan Degradation	0.5571	0.1321	0.4710	0.1614	0.083	0.4714	0.1573	0.4704	0.1796	0.694
Glycolysis	1.2758	0.2126	1.3462	0.2524	0.274	1.2953	0.1916	1.4536	0.3365	0.350
Inorganic Nutrient Metabolism	0.5488	0.1478	0.5722	0.2681	0.626	0.6120	0.2579	0.4883	0.2851	0.302
Metabolic Regulator Biosynthesis	0.0487	0.0589	0.0842	0.0919	0.131	0.1029	0.1018	0.0447	0.0511	0.115
Nucleic Acid Processing	0.3746	0.1368	0.4136	0.1256	0.303	0.3927	0.1259	0.4575	0.1199	0.302
Nucleoside and Nucleotide Biosynthesis	11.1007	0.8553	11.2926	1.9080	0.756	10.7594	1.1346	12.4182	2.6999	0.077

Nucleoside and Nucleotide Degradation	1.9739	0.6383	1.9665	0.4026	0.560	1.9763	0.3898	1.9458	0.4522	0.922
Amine and Polyamine Biosynthesis	0.3397	0.1601	0.3123	0.1899	0.367	0.3328	0.2045	0.2691	0.1567	0.403
Other	27.9648	1.4064	28.5770	1.2494	0.109	28.5324	1.3563	28.6712	1.0556	0.883
Other Biosynthesis	0.1928	0.0985	0.2232	0.1067	0.288	0.2438	0.0879	0.1796	0.1336	0.115
Pentose Phosphate Pathways	0.9656	0.2003	0.8850	0.1035	0.080	0.8976	0.1118	0.8585	0.0827	0.350
Photosynthesis	0.5930	0.0858	0.5692	0.0786	0.274	0.5676	0.0865	0.5727	0.0631	0.658
Polymeric Compound Degradation	1.3427	0.2637	1.1972	0.3503	0.116	1.2162	0.2896	1.1570	0.4719	0.658
Protein Modification	0.0000	0.0000	0.0000	0.0000	0.895	0.0000	0.0000	0.0000	0.0000	0.157
Respiration	0.0456	0.0565	0.0432	0.0516	0.792	0.0344	0.0374	0.0616	0.0726	0.491
Secondary Metabolite Biosynthesis	1.9945	0.1495	1.8962	0.2544	0.078	1.9039	0.2154	1.8798	0.3370	0.806
Secondary Metabolite Degradation	1.5897	0.4035	1.6315	0.5838	0.708	1.6628	0.4840	1.5655	0.7848	0.077
TCA cycle	1.2021	0.4657	1.2820	0.4768	0.696	1.3665	0.4611	1.1036	0.4859	0.140
Amine and Polyamine Degradation	0.2746	0.1046	0.2790	0.1387	0.994	0.3052	0.1322	0.2239	0.1433	0.085
Amino Acid Biosynthesis	12.5705	1.5203	11.7091	2.7192	0.020	12.0428	2.7029	11.0046	2.7738	0.922
Amino Acid Degradation	0.2432	0.1426	0.3844	0.2393	0.017	0.4185	0.2604	0.3124	0.1790	0.376
Aminoacyl-tRNA Charging	0.4975	0.0772	0.4534	0.1063	0.080	0.4565	0.0980	0.4467	0.1284	0.491
Antibiotic Resistance	0.2654	0.0977	0.2262	0.2327	0.029	0.1865	0.0848	0.3100	0.3936	0.806
Aromatic Compound Biosynthesis	1.0224	0.1632	0.9518	0.1738	0.056	0.9320	0.1869	0.9937	0.1428	0.238

Abbreviations: ESRD, end stage renal disease; PD, peritoneal dialysis; PNCI, peritoneal dialysis patient with normal cognition; PCI, peritoneal dialysis patient with cognitive impairment.
