# Supplementary material for: Correlation between gut microbiome and cognitive impairment in patients undergoing peritoneal dialysis
Source: BMC Nephrol. 2023 Dec 5;24:360. doi: 10.1186/s12882-023-03410-z (PMC10696889; doi:10.1186/s12882-023-03410-z)
Supplement: Supplementary file 11 — Additional file 11: Table S10. The clinical detail of each patient in PD and ESRD groups. [file 12882_2023_3410_MOESM11_ESM.pdf]

Table S10. The clinical detail of each patient in PD and ESRD groups.

Serial number	Group	Age (Years)	Sex (M /F)	Education (Years)	BMI (kg/m2)	eGFR [ml/min·1.73 m2]	MMSE	MoCA	SAS	SDS	HAMA	HAMD	GSRS
1	ESRD	38	female	12	28.72	7	29	27	34	25	3	1	24
2	ESRD	52	male	5	21.88	10	27	18	23	28	15	14	20
3	ESRD	37	female	6	29.73	7	29	27	34	42	9	6	24
4	ESRD	54	male	8	23.03	8	29	25	31	39	17	10	29
5	ESRD	56	female	2	18.75	3	26	21	26	33	16	14	27
6	ESRD	44	female	7	26.30	9	27	21	26	33	13	14	21
7	ESRD	52	male	10	23.26	6	27	29	36	45	13	4	18
8	ESRD	45	female	8	22.48	4	29	26	33	41	6	2	25
9	ESRD	53	male	7	24.77	8	29	22	28	51	22	12	23
10	ESRD	39	female	5	20.08	4	28	27	34	42	13	3	29
11	ESRD	31	female	8	21.45	9	26	23	29	55	14	11	20
12	ESRD	55	male	17	23.99	7	30	27	34	42	3	1	21
13	ESRD	55	male	2	25.95	8	24	18	23	28	10	11	20
14	ESRD	31	female	12	17.15	9	28	25	31	61	12	8	15
15	ESRD	50	male	5	23.44	9	30	25	31	30	5	2	18
16	ESRD	45	male	11	26.47	8	29	28	35	39	6	3	20
17	ESRD	39	male	7	28.40	6	28	25	31	41	10	7	21

18	ESRD	34	male	8	21.39	5	29	26	33	40	3	2	17
19	ESRD	43	female	8	16.82	9	28	28	35	35	14	5	24
20	ESRD	34	female	8	28.76	12	26	28	35	28	4	4	19
21	ESRD	41	male	15	24.51	7	29	27	34	46	16	10	17
22	ESRD	43	male	8	24.91	5	30	28	35	44	4	2	22
23	ESRD	49	male	8	26.00	7	29	27	34	42	14	11	20
24	ESRD	58	male	8	18.61	9	27	28	35	35	11	6	25
25	ESRD	53	male	14	23.67	9	29	28	35	39	11	6	19
26	ESRD	39	female	12	18.32	7	26	27	34	29	6	3	16
27	ESRD	43	female	15	22.43	6	30	27	34	39	10	10	21
28	ESRD	45	female	11	24.84	9	28	24	30	38	6	7	26
29	ESRD	54	male	5	23.88	6	29	25	31	39	17	12	25
30	PD	58	female	6	21.09	4	24	21	60	61	24	18	31
31	PD	50	male	1	26.08	3	25	18	34	43	10	8	27
32	PD	42	female	8	20.34	4	27	23	35	39	8	3	27
33	PD	42	female	8	18.36	4	26	24	39	30	8	5	27
34	PD	58	male	11	22.13	3	29	25	34	35	13	8	25
35	PD	50	female	6	18.87	2	22	18	29	30	10	5	21
36	PD	43	male	5	26.57	3	24	18	33	34	9	7	21
37	PD	64	male	6	27.34	3	28	22	31	35	7	4	22
38	PD	47	male	6	23.46	6	27	20	31	43	8	6	21

39	PD	56	female	1	21.48	4	16	9	36	41	8	9	26
40	PD	50	male	3	21.67	7	21	16	29	25	0	2	21
41	PD	55	female	10	21.09	3	29	22	29	36	8	5	24
42	PD	52	female	12	23.71	3	28	24	36	31	11	11	22
43	PD	55	male	7	22.99	4	28	24	36	34	6	5	20
44	PD	35	female	9	22.55	3	23	20	28	26	3	1	19
45	PD	48	male	7	23.84	4	27	20	29	29	2	4	26
46	PD	40	male	6	20.18	4	25	21	43	56	17	9	20
47	PD	56	female	3	21.76	4	29	23	36	35	13	6	25
48	PD	55	female	3	23.63	4	23	14	39	38	12	7	20
49	PD	49	female	8	20.81	5	27	28	39	34	6	3	18
50	PD	38	male	8	27.18	3	30	28	40	38	14	8	29
51	PD	34	female	8	17.58	4	28	27	45	48	9	9	29
52	PD	33	female	8	22.27	4	28	26	26	29	3	1	22
53	PD	57	male	8	17.37	5	27	29	39	50	8	8	20
54	PD	25	female	8	21.36	7	28	27	30	29	4	3	26
55	PD	40	female	9	23.53	8	30	26	31	33	4	3	26
56	PD	18	male	12	16.00	4	30	29	38	39	11	4	20
57	PD	31	male	8	17.72	5	29	26	33	34	3	2	26

Abbreviations: ESRD, end stage renal disease; PD, peritoneal dialysis; BMI, body mass index; eGFR, estimated glomerular filtration rate; MoCA, Montreal cognitive assessment scale; MMSE, Mini-mental state examination; SAS, Self-rating anxiety scale; SDS, Self-rating depression scale; HAMA, Hamilton anxiety scale; HAMD, Hamilton depression scale; GSRS, Gastrointestinal symptom rating scale.
